# LiDFOB Initiated In Situ Polymerization of Novel Eutectic Solution Enables Room‐Temperature Solid Lithium Metal Batteries

**DOI:** 10.1002/advs.202003370

**Published:** 2020-11-03

**Authors:** Han Wu, Ben Tang, Xiaofan Du, Jianjun Zhang, Xinrun Yu, Yantao Wang, Jun Ma, Qian Zhou, Jingwen Zhao, Shanmu Dong, Gaojie Xu, Jinning Zhang, Hai Xu, Guanglei Cui, Liquan Chen

**Affiliations:** ^1^ Qingdao Industrial Energy Storage Research Institute Qingdao Institute of Bioenergy and Bioprocess Technology Chinese Academy of Sciences Qingdao 266101 China; ^2^ College of Chemistry and Chemical Engineering Central South University Changsha 410083 China; ^3^ Beijing National Laboratory for Condensed Matter Physics Institute of Physics Chinese Academy of Sciences Beijing 100190 China

**Keywords:** eutectic solution, in situ polymerization, room‐temperature lithium metal batteries, solid‐state polymer electrolytes

## Abstract

It is demonstrated that a novel eutectic solution including 1,3,5‐trioxane (TXE) and succinonitrile (SN) can be converted into solid‐state polymer electrolyte (SPE) via in situ polymerization triggered by lithium difluoro(oxalato)borate (LiDFOB). It is worth noting that all the precursors (LiDFOB, TXE, and SN) of this novel SPE are totally solid and nonvolatile at room temperature, where, LiDFOB works as a lithium salt and an initiator simultaneously to avoid the introduction of impurity. It is noted that such SPE presents a considerable ionic conductivity of 1.14 × 10^−4^ S cm^−1^ and a sufficiently wide electrochemical window of 4.5 V, which is significant for supporting the high‐energy lithium batteries. In addition, this dedicatedly designed in situ polymerization is powerful to build kinetically favorable polymer‐based protective layers on LiCoO_2_ cathode and Li metal anode simultaneously, guaranteeing outstanding cycling stability (capacity retention of 88% after 200 cycles) of 4.3 V LiCoO_2_/lithium metal batteries at room temperature. More intriguingly, soft packed LiCoO_2_/SPE/Li metal batteries can still light a blue light emitting diode (LED) under the harsh conditions of being bent, cut, and stroked by a hammer, demonstrating excellent safety characteristics.

## Graphical abstract

A new type of solid‐state polymer electrolyte (SPE) free of additional initiators and flammable precursors is dedicatedly designed through an in situ polymerization of novel eutectic solution by LiDFOB. Such SPE can build kinetically favorable protective layers on LiCoO_2_ and Li simultaneously, guaranteeing outstanding cycling stability of 4.3 V LiCoO_2_/Li metal battery.

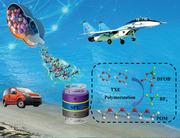

In situ generation of solid‐state polymer electrolytes (SPEs) has attracted ever‐increasing attention in terms of their flexibility in large‐scale production and ameliorated interfacial resistance originating from their well‐matched lithium ion battery technology and favorable interfacial integration.^[^ ^1^ ^]^ It is noted that in situ generated SPEs by polymerization of precursor especially mobile monomer has been approved as an effective approach to maintain superior interfacial compatibility and enhance room‐temperature ionic conductivity of SLBs.^[^ ^2^ ^]^ However, most of in situ formed SPEs in previous reports still have unsolved intrinsic defects. For instance, it is often necessary to introduce additional initiators like azobis(isobutyronitrile) (AIBN) to initiate the free radical polymerization,^[^ ^3^ ^]^ which may react with electrodes and then have negative effect on electrochemical performances of the as‐assembled SLBs. Furthermore, the precursors of these reported in situ generated SPEs often contain volatile and flammable solvent.^[^ ^4^ ^]^ Therefore, the unpolymerized liquid precursors or solvent will remain in SPEs, which have the great possibility to trigger safety risks. Thus, it is urgent to develop the in situ formed SPEs with no presence of additional initiators and volatile, flammable residues.

1,3,5‐trioxane (TXE), a common industrial material with a melting point of 65 °C,^[^ ^5^ ^]^ is one of the most accessible and cheap raw material for the synthesis of poly(formaldehyde) (POM). In addition, POM exhibits similar structure to poly(ethylene oxide) (PEO) and favorable outer electronic configuration, which is a potential candidate for SPEs. However, it tends to form highly crystalline and high molecular weight engineering polymer during the in situ polymerization if initiated by catalyst, which is unfavorable to present high ionic conductivity.^[^ ^6^ ^]^ Succinonitrile (SN), a retarded agent of polymerization, which can influence corresponding molecular weight and following lessen crystallinity, is introduced to form a eutectic solution with TXE and improve ionic conductivity of the in situ generated SPE. More importantly, TXE and SN can form a eutectic solution due to the interaction between them and thereby facilitate the fabrication procedure of batteries.

Herein, we dedicatedly design a new type of SPE free of additional initiators (LiDFOB works as lithium salt and initiator simultaneously) and liquid precursors (LiDFOB, TXE, and SN are totally solid and non‐volatile at room temperature) through an in situ polymerization of novel eutectic solvent including TXE and SN initiated by LiDFOB. Compared with most of previously reported in situ strategies,^[^ ^3^, ^7^ ^]^ our case is well designed with superb advantages including free of extra initiators, cheap raw materials, and nonvolatile precursors at room temperature.

Furthermore, this as‐designed SPE is advantageous to generate favorable protection layers on LiCoO_2_ cathode and Li metal anode with considerable conductivity of 1.14 × 10^−4^ S cm^−1^ at room temperature, and thus enabling superior cycle performance (capacity retention of 88% after 200 cycles at room temperature) for 4.3 V LiCoO_2_/Li metal batteries. Moreover, even at the active material mass loading of 11.5 mg cm^−2^, it can still deliver a considerable areal capacity of 1.72 mAh cm^−2^ (initial areal capacity is 1.75 mAh cm^−2^) at room temperature. Besides, when used in lithium metal batteries, TXE prefers to preferentially generate protective polymer layer on the surface of Li metal and then effectively prevents the parasitic side‐reactions between SN and Li metal owing to the polymerization of TXE, which is advantageous to improve interfacial compatibility. It is also worthy to note that, due to the absence of volatile residues and flammable solvent, soft packed LiCoO_2_/SPE/Li metal batteries are capable of powering blue light‐emitting diode (LED) in the harsh conditions of being bent, cut and stroked by a hammer, demonstrating fantastic safety characteristics of this as‐developed SLBs. Meanwhile, the well‐designed SPE endows 4.3 V LiNi_0.6_Co_0.2_Mn_0.2_O_2_/Li metal batteries superior cycling stability (capacity retention of 83.5% after 170 cycles) and high coulombic efficiency (99%) at room temperature. The present study opens up a new avenue in boosting room‐temperature SLBs and also enlightens the research and development of other high‐performance metal batteries.

Figure S1 in the Supporting Information displays the physical state of three raw materials (SN, TXE, and LiDFOB) of SPE at room temperature. SPEs contained various mass ratios of TXE:SN (5:1, 5:2, 5:3, 5:4, and 5:5), which are abbreviated as PSL51, PSL52, PSL53, PSL54, and PSL55, respectively. When the mass ratios of TXE and SN are 5:1 and 5:2, they have relatively low ionic conductivities at room temperature (Figure S15, Supporting Information). Furthermore, according to Table S1 (Supporting Information), at the mass ratios of 5:4 and 5:5, the molecular weights of PSLs are too low. As a result, PSL53 was chosen for following researches. **Figure** **1**a displays the fabrication of in situ generated PSL53. Due to the interaction between the lone pair electrons on the oxygen of TXE and the strong electron‐absorbing cyano group on SN, desired amount of TXE and SN can be mixed to obtain eutectic solution (Figure S2, Supporting Information). Then, LiDFOB is added into the obtained eutectic solution and initiated the in situ polymerization of TXE at 80 °C to generate SPE. In addition, the proposed polymerization process of TXE is schemed in Figure 1b. LiDFOB first decomposes into lithium tetrafluoroborate (LiBF_4_) and lithium bis(oxalato)borate (LiBOB) on the surface of lithium metal with the aid of alkaline lithium.^[^ ^8^ ^]^ Following, LiBF_4_ decomposes into BF_3_ and LiF,^[^ ^9^ ^]^ (the existence of BF_3_ has been approved by X‐ray photoelectron spectroscopy (XPS) in Figure S3 in the Supporting Information) where BF_3_ works as an initiator to trigger the open‐ring polymerization of TXE at 80 °C. BF_3_ initially attacks the oxygen atom and induces TXE monomers to transform into the reactive second oxonium ions via fast protonation, and then the repetitious interposition of TXE into the oxonium ions results in the growth of polymer chains. (The polymerization of TXE has been proved by NMR, FTIR, and Raman in Figures S4–S6 in the Supporting Information).

**Figure 1:**
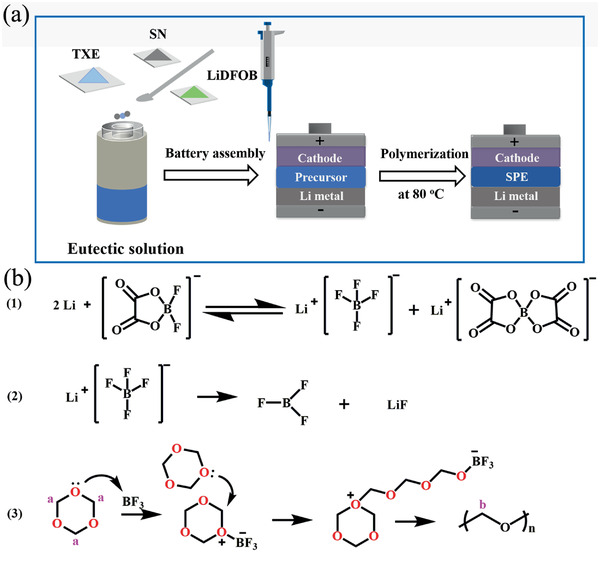
a) Schematic representation of the in situ generated SPE process. b) Possible polymerization process of TXE.

It is well recognized that thermal characteristic of SPE is critical for safety performance of SLB.^[^ ^10^ ^]^ The physical states of SPE have been shown in Figures S7 and S8 in the Supporting Information. The PSL53 can maintain its solid state even at an elevated temperature (i.e., 80 °C). Then, differential scanning calorimetry (DSC) is used to evaluate the thermal characteristic of PSL53. As shown in Figure S9 (Supporting Information), the melting point (*T*_m_) of SN is typically around 55 °C, indicating SN‐LiDFOB will turn into a fluid when the temperature is higher than 55 °C. In contrast, the *T*_m_ of SN in PSL53 vanishes, meaning that SN has a strong interaction with POM, which generates completely uniform super‐molecular structure, rather than merely physical mixing. We believed that, the super‐molecular structure is formed by the interaction between the lone pair electrons on the oxygen of POM and the strong electron‐absorbing cyano group on SN. We have conducted the theoretical calculation, solid state NMR and IR to verify the interaction. The snapshots of the three components mixture system (LiDFOB, SN, POM) from classical molecular dynamics (MD) simulations are shown in **Figure** **2**a. First, they clearly demonstrate that Li molecules are closely coordinated with POM instead of SN because there is obvious peak of the Li‐POM pair in 3.4 Å.^[^ ^11^ ^]^ Furthermore, a peak of the POM‐SN pair (5.1 Å) is identified in this system, which means that there is a strong interaction between POM and SN (Figure 2b). The solid state NMR gives strong support to the MD summations. As is shown in Figure 2c, after mixing with SN, the chemical shift of POM moves from 4.721 to 5.210 ppm. At the same time, the chemical shift of SN moves from 2.956 to 2.860 ppm. These changes of chemical shift in the NMR represent the changes of electron clouds, meaning that the electron cloud exchange occurs between POM and SN after the two molecules mixed. Besides, the IR results also verified the interaction between POM and SN (Figure S10, Supporting Information). Furthermore, as shown in Figure S11 (Supporting Information), the binding energy obtaind from density functional theory (DFT) calculations between POM and SN is −0.4638 eV which is higher than that of TXE and SN (−0.1703 eV), corroborating that SN are more likely to bind to POM and form a uniform super‐molecular structure. By forming a super‐molecular structure with SN, POM can break the interaction between TXE and SN to retain the solid state of PSL53. Furthermore, as shown in Figure 2d, this structure can suppress the motion of SN and prevent SN from directly contacting with Li metal (SN can react with Li metal and thus deteriorate cycling stability of batteries).

**Figure 2:**
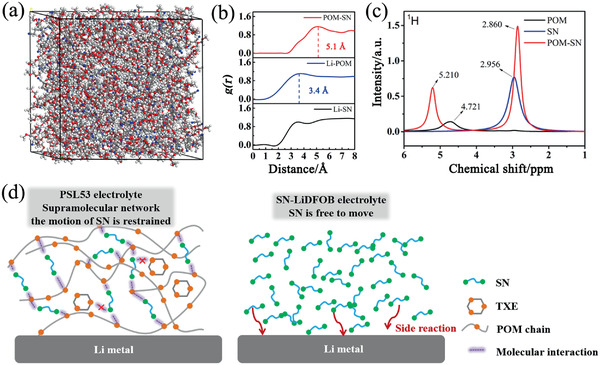
a) MD simulation snapshots of LiDFOB, POM, and SN mixture. b) Radial distribution functions g(r) of Li‐SN, Li‐POM, and POM‐SN pairs calculated from MD simulation trajectories at 353 K. c) ^1^H MAS NMR of POM, SN, and POM‐SN mixrture. d) Comparsion of PSL53 and SN‐LiDFOB electrolyte.

Linear sweep voltammetry (LSV) analysis of the Stainless steel/Li cells at a low scan rate of 1 mV s^−1^ reveals a weak current between 3.0 and 5.5 V in battery using SN‐LiDFOB, before an exponential increase is apparent (Figure S12, Supporting Information). As SN is known to be stable in this voltage range, it is speculated that the current peak is related to the reactions between SN and Li metal. In comparison, the curve is much more stable in battery using PSL53, indicating that the reactions between SN and Li metal have been suppressed in such battery. The electronic conductivities of electrolytes have also been tested in Figure S13 in the Supporting Information. The electronic conductivity of PSL53 is calculated as 2.7 × 10^−8^ S cm^−1^, which is low enough for lithium batteries.^[^ ^12^ ^]^ Besides electronic conductivity, ionic conductivity is a much more important parameter to evaluate the performance of electrolyte. As illustrated in Figure S15a (Supporting Information), the ionic conductivity of PSL53 approaches a value of 1.14 × 10^−4^ S cm^−1^ at room temperature. Although it is lower than that (7.74 × 10^−4^ S cm^−1^) of SN‐LiDFOB, it can still merit in SLB at room temperature. We also find that SN can reduce the crystallinity of PEO and enhance ionic conductivity. As shown in Figure S14 (Supporting Information), with the increase of the ratio of SN, the crystallinity of the polymer decreases gradually. At the same time, the ionic conductivity of the SPE increase (Figure S15b, Supporting Information). We also use ^7^Li solid state NMR spectra to indentify mobile and immobile cations in these two electrolytes (Figure S16, Supporting Information). Li/Li symmetric cells were assembled and used to investigate the interfacial compatibility of electrolytes (SN‐LiDFOB and PSL53) with Li metal anode (Figure S17, Supporting Information). It is obvious that PSL53 displays a much favorable electrochemical performance than that of SN‐LiDFOB.

The electrochemical performance of 4.3 V LiCoO_2_/Li metal batteries with varied electrolytes have been compared at room temperature and −10 °C. It is worth noting that the polarization of 4.3 V LiCoO_2_/PSL53/Li metal battery (**Figure** **3**b) is much lower than that of SN‐LiDFOB (Figure 3a). In addition, as shown in Figure S18 (Supporting Information), CV curves of 4.3 V LiCoO_2_/PSL53/Li metal battery are much more symmetrical and rational than that of the counterpart. As shown in Figure 3c, 4.3 V LiCoO_2_/SN‐LiDFOB/Li metal battery undergoes an abrupt capacity decay from 35 mA h g^−1^ to nearly 0 after 20 cycles. Such rapid capacity fading is caused by the fact that SN reacts with Li metal and thereby results in fast battery failure. In a sharp contrast, 4.3 V LiCoO_2_/PSL53/Li metal battery delivers superior cycle performance (capacity retention of 88% after 200 cycles) and high Coulombic efficiency (99.3%) at room temperature, which are much better than those of SN‐LiDFOB. It is noted that the as‐prepared SLB can also tolerate relatively low temperatures for wide application especially in cold environment. As presented at Figure 3d, even at relatively low temperature of −10 °C, the as‐assembled 4.3 V LiCoO_2_/PSL53/Li metal battery delivers satisfactory discharge specific capacity of 120.5 mAh g^−1^ at the first cycle and then gradually increase to 140.5 mA h g^−1^ after 35 cycles. To the best of our knowledge, few SLBs can operate with a considerable performance at low temperatures (Table S2, Supporting Information). This further proves the superiority of PSL53 electrolyte over its counterpart previously reported.

**Figure 3:**
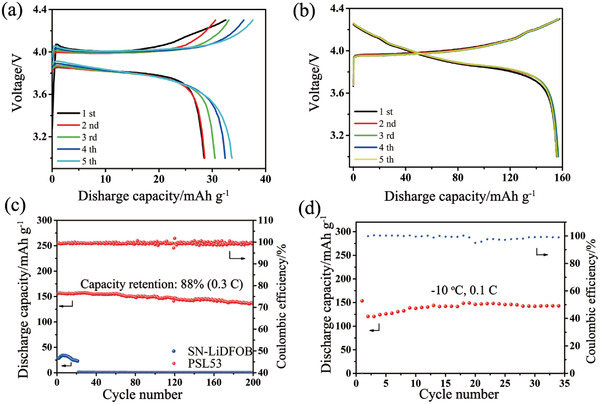
a) Charge/discharge profiles of 4.3 V LiCoO_2_/SN‐LiDFOB/Li metal cell at the rate of 0.3 C at room temperature. b) Charge/discharge profiles of 4.3 V LiCoO_2_/PSL53/Li metal battery at the rate of 0.3 C at room temperature. c) Cycle performance of 4.3 V LiCoO_2_/Li using SN‐LiDFOB and PSL53 at the rate of 0.3  C at room temperature. d) Cycling stability of 4.3 V LiCoO_2_/PSL53/Li metal battery with the rate of 0.1 C at −10 °C.

As far as we know, most of previously reported state‐of‐the‐art SLBs using in situ generated SPE mainly employed low areal active material loadings cathodes (≤5 mg cm^−2^) owing to a formidable obstacle of ionic diffusion (Table S2, Supporting Information). However, the cathode with high mass loadings coupling Li metal is indispensable for developing high‐energy lithium metal batteries. Hence, we further investigate the rate capability and cycle performance of 4.3 V LiCoO_2_/PSL53/Li metal batteries at varied areal active material loadings (1.0, 3.7, 6.7, and 11.5 mg cm^−2^). As demonstrated in Figure S19 (Supporting Information), even at a relatively high current density of 1 C, only minor fading of areal capacity can be spotted in varied areal active material loadings. Apparently, for the LiCoO_2_ cathode of 6.7 mg cm^−2^ at the rate of 1 C, it can still deliver a discharge areal capacity of 0.89 mAh cm^−2^ (equal to 80% of that at 0.3 C), manifesting the excellent rate capability for 4.3 V LiCoO_2_/PSL53/Li metal battery. At a high mass loading of 5.8 mg cm^−2^ and current density of 0.1 C, 4.3 V LiCoO_2_/PSL53/Li metal battery can maintain a capacity retention of 89% after 180 cycles (Figure S20, Supporting Information). Furthermore, in Figure S21 (Supporting Information), even at a high mass loading of 11.5 mg cm^−2^, 4.3 V LiCoO_2_/PSL53/Li metal battery can still deliver a favorable areal capacity of 1.72 mA h cm^−2^ even after 45 cycles (initial areal capacity is 1.75 mA h cm^−2^). Furthermore, we also use the highest cathode loading of 18.58 mg cm^−2^, which can be achieve by our current cathode fabricated processes for the electrochemical tests. As shown in Figure S22 (Supporting Information), at a relatively low rate of 0.05 C, the battery using PSL53 electrolyte can achieve a high cathode capacity of ≈3 mAh cm^−2^. This superior cycling stability of 4.3 V LiCoO_2_/PSL53/Li metal batteries should be ascribed to the excellent interfacial compatibility, arising from the protective role of layers formed on LiCoO_2_ cathode and Li metal anode respectively.

Transmission electron microscopy (TEM) measurement shows that there is a passivation layer on the surface of LiCoO_2_ cathode cycled with PSL53 (Figure S24, Supporting Information). Simultaneously, scanning electron microscopy (SEM) and energy dispersive X‐ray spectroscopy (EDS) mapping of cycled LiCoO_2_ cathode are applied to reveal its morphology and elemental distribution of this layer. As shown in **Figure** **4**a,b, in the cathode cycled with SN‐LiDFOB, there are broken strips shown on the surface of LiCoO_2_ particles. Furthermore, the Co and O have strong signals and show the same distribution patterns, indicating that there is no surface layer on LiCoO_2_ particles. In sharp comparison, the surface of LiCoO_2_ cycled with PSL53 has a relatively smooth surface. Furthermore, the C, O, F, and Co elements are showing the same distribution patterns, while the Co signals are relatively sparse. We believe that this phenomenon is caused by in situ formed passivation layer covered over LiCoO_2_ particles and block the signal of Co. Figure S24 (Supporting Information) depicted typical TEM images of cycled LiCoO_2_ cathodes disassembled from 4.3 V LiCoO_2_/Li metal batteries with SN‐LiDFOB and PSL53 after cycling. Obviously, a well‐distributed passivation layer formed on the surface of LiCoO_2_ with a thickness of about 15 nm when using PSL53, which is in sharp contrast with that (no obvious layer) of LiCoO_2_ cathodes disassembled from LiCoO_2_/SN‐LiDFOB/Li metal battery. This passivation layer is possibly formed by the in situ polymerization of TXE, which can suppress the dissolution of Co and maintain the structure integration of LiCoO_2_ particles during long‐term cycles, which can be proved by SEM result of Figure S25 in the Supporting Information.

**Figure 4:**
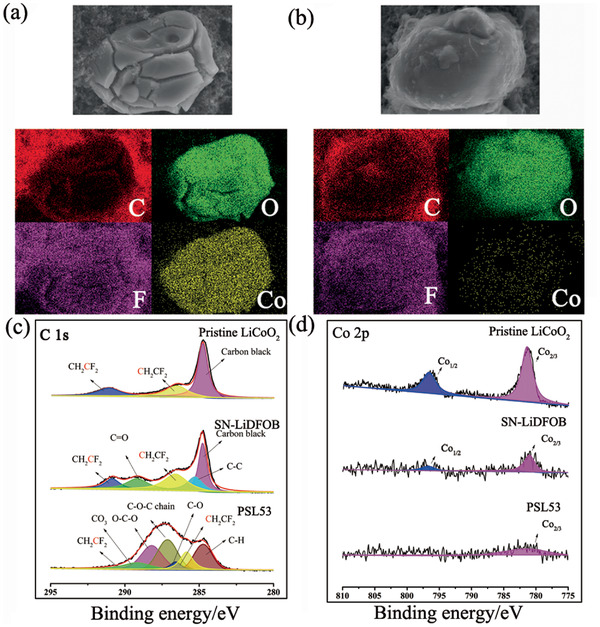
SEM images and EDS mapping results of a) LiCoO_2_ cathode cycled with SN‐LiDFOB b) LiCoO_2_ cathode cycled with PSL53. c) C 1s and d) Co 2p XPS spectra of cycled LiCoO_2_ cathodes disassembled from 4.3 V LiCoO_2_/Li metal batteries using SN‐LiDFOB and PSL53 after cycling.

XPS is used to further characterize the chemical composition of the layer on the surface of cycled LiCoO_2_ cathodes. As shown in Figure 4c, the XPS spectra (284.6, 286.4, and 291.2 eV belonging to carbon black and PVDF) of LiCoO_2_ cathode cycled with SN‐LiDFOB is similar to that of pristine LiCoO_2_ electrode.^[^ ^13^ ^]^ except signal belonging to LiDFOB (C—C, 285.27 eV) and decomposition product (C=O, 289.2 eV) of LiDFOB. By contrast, the major peaks (284.7, 286.9, and 288.2 eV) of LiCoO_2_ cathode collected from PSL53‐based battery are ascribed to POM.^[^ ^14^ ^]^ This finding can also be corroborated by the Co 2p. As displayed in Figure 4d, the Co 2p_3/2_ peaks at 781.3 and 799.8 eV are the characteristic peaks of LiCoO_2_ cathode.^[^ ^15^ ^]^ These peaks in the spectra of LiCoO_2_ cathode collected from PSL53‐based battery are much weaker than those of LiCoO_2_ cathode collected from SN‐LiDFOB electrolyte‐based battery, indicating that a layer was generated on the surface of LiCoO_2_ and block the signal of Co.

The F 1s spectra reflected in Figure S26 (Supporting Information) also provides valuable information about this passivation layer. Concretely, the spectra of pristine LiCoO_2_ cathode and LiCoO_2_ cathode cycled with SN‐LiDFOB both have strong peak belonging to PVDF (688.2 eV). Besides, on the surface of LiCoO_2_ cycled with SN‐LiDFOB, there are strong signals belonging to the decomposition products (B–F, 685.85 eV, BF_3_ 686.77 eV) of LiDFOB.^[^ ^16^ ^]^ In comparison, a much stronger characteristic peak of LiF (685 eV) and weaker signals belonging to other decomposition products (B–F, BF_3_) of LiDFOB and PVDF appear on the surface of LiCoO_2_ cathode cycled with PSL53. This phenomenon manifests that LiF, a beneficial component of the stable passivation layer for high‐voltage cathodes,^[^ ^17^ ^]^ is more likely to form on the surface of cathode when cycled with PSL53. Combining the results of TEM, SEM, and XPS, we have reached a conclusion that this passivation layer (mainly consists of POM and LiF) formed on the surface of LiCoO_2_ cathode, which can effectively maintain the structure integration of LiCoO_2_.

Besides the effective passivation layer on cathodes, interfacial compatibility between SPE and Li metal anode is another problem need to be solved for high‐energy lithium metal batteries. It is noted that SN has been reported to react with Li metal and thereby results in dramatic battery failure.^[^ ^18^ ^]^ However, as mentioned above, PSL53‐based battery exhibits outstanding cycle performance. We speculate that PSL53 may incline to form a protective layer on the surface of Li metal and then favorably suppress the parasitic side‐reactions between SN and Li metal.^[^ ^19^ ^]^ **Figure** **5**a depicts typical SEM images of cycled Li metal anodes disassembled from 4.3 V LiCoO_2_/Li cells with SN‐LiDFOB and PSL53 after cycling. Obviously, mossy electrodeposits can be detected on the surface of Li metal dismantled from SN‐LiDFOB based battery. Furthermore, as shown in Figures S27 and S28 (Supporting Information), there is serious erosion in the cross‐section SEM image of lithium metal anode collected from SN‐LiDFOB based battery. It can be assumed that these structures are induced by the parasitic side‐reactions between SN and Li metal.^[^ ^20^ ^]^ In sharp comparison, the surface of Li metal dismantled from PSL53 based batteries visibly flat even after 200 cycles (Figure 5b). In addition, a relatively smooth surface is also found in the cross‐section SEM image of lithium metal anode. These results further prove that PSL53 can suppress the side‐reactions between SN and Li metal, and thus enhance interfacial compatibility.

**Figure 5:**
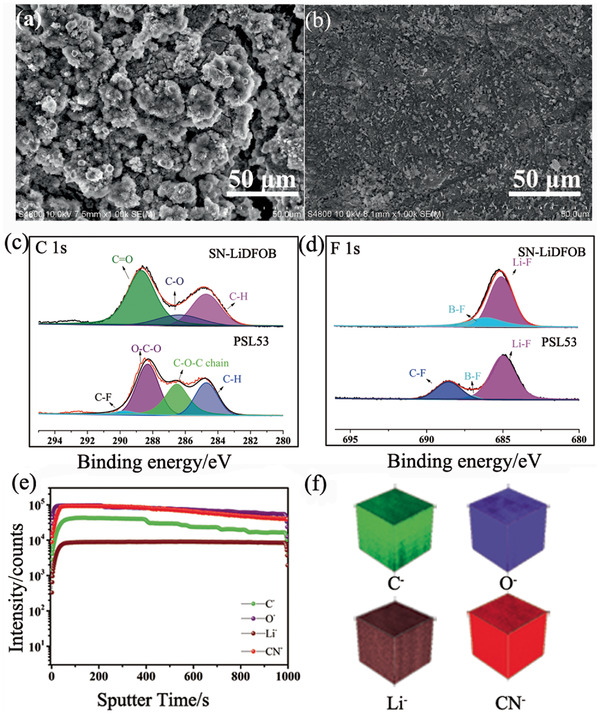
Typical SEM images of cycled Li metal anodes disassembled from 4.3 V LiCoO_2_/Li metal cells with a) SN‐LiDFOB and b) PSL53. XPS spectra of c) C 1s and d) F 1s of cycled Li metal anodes disassembled from 4.3 V LiCoO_2_/Li metal batteries with SN‐LiDFOB and PSL53. e) TOF‐SIMS negative ion depth profiles and f) compiled 3D diagram of cycled Li metal anode disassembled from 4.3 V LiCoO_2_/PSL53/Li metal batteries.

To further explore the chemical composition of this protective layer, cycled Li metal anodes disassembled from LiCoO_2_/Li metal batteries are further characterized by XPS. It is clearly seen in Figure 5c that C—H (284.7 eV), C—O (286.3 eV), and C=O (288.7 eV)^[^ ^21^ ^]^ of cycled Li metal anode disassembled from LiCoO_2_/SN‐LiDFOB/Li metal batteries cycled are attributed to the decomposition products of LiDFOB. In contrast, new peaks (286.9 and 288.2 eV)^[^ ^14^ ^]^ corresponding to POM are also detected on the surface of Li metal cycled with PSL53, demonstrating that the protective layer on the surface of Li metal contains POM. Moreover, compared to the F 1a spectrum of Li metal surface with SN‐LiDFOB which have only one peak belonging to Li—F (685 eV), additional peaks of B—F (686.7 eV), C—F (688.5 eV)^[^ ^22^ ^]^ appeared on the Li metal surface with PSL53 (Figure 5d).

Time‐of‐flight secondary‐ion mass spectrometry (TOF‐SIMS) in‐depth analysis reveals the distribution of the components in the deeper layer. Apparently, C^−^, O^−^, CN^−^, and Li^−^ fragments are uniformly distributed (Figure 5e,f), indicating that POM, SN, and LiDFOB show a uniform distribution in this protective layer on the surface of lithium metal. Moreover, this layer almost has the same component as PSL53, which is favorable for lithium ion conduction. However, if SN directly contact with Li metal, it will react with Li metal and cause dramatic capacity fading. We assumed that TXE tended to form stable protecting layer mainly consisting POM on the surface of Li metal owing to the polymerization of TXE induced by the preferential decomposition products of LiDFOB, and thus, protected against side‐reactions between Li metal and SN.

In order to gain further insight into the chemical stability of this protective layer with lithium metal, the quantum chemical calculation based on DFT are used to evaluate its thermodynamic stability. As displayed in Figure S29 (Supporting Information), the DFT calculations reveal that LiDFOB has the lowest LUMO energy of the three components of our electrolyte (LiDFOB, SN, POM), hence it is preferentially to be reduced on Li metal. It is a common knowledge that LiDFOB is reduced to produce BF_3_ at the surface of Li metal, which initiated the polymerization of TXE on the surface of Li metal. As shown in **Figure** **6**a, the first‐principle calculations indicate that the interface interaction energy (−1.52 eV) of POM in Li‐110 surface is higher than that of SN (−1.06 eV) and DFOB^−^ (−0.95 eV, Figure S30, Supporting Information). The obtained DFT calculations results predict that POM will preferentially adhere to lithium (Figure 6b). Such in situ generated thin POM layer at Li metal surface with stable LUMO energy level is compatible with Li metal anode. Thus, this thin POM layer can effectively protect side‐reactions between Li metal and SN and thus improve interfacial compatibility between PSL53 and Li metal. In addition, POM possesses a high Young's modulus (6 GPa),^[^ ^1a^ ^]^ which is advantageous to suppress the growth of Li dendrites.

**Figure 6:**
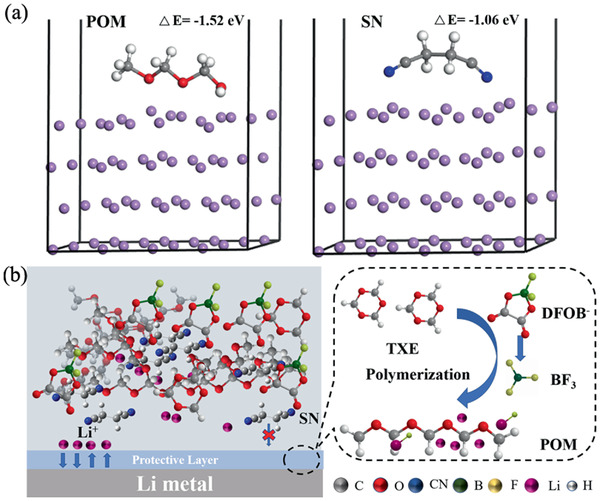
a) The density functional theory (DFT) calculations on the interface interaction energy between the molecules (POM, SN) and Li‐110 slab. b) The proposed formation mechanism of protective layer on the surface of Li metal.

Based on the battery performance and postmortem analysis, we propose multi‐functional mechanism of PSL53 in 4.3 V LiCoO_2_/Li metal batteries, consisting of: (1) Generating an effective passivation layer (mainly consists of POM and LiF) on LiCoO_2_ cathode to suppress the dissolution of Co. (2) Forming a protective layer mainly composed of POM on the surface of Li metal to prevent side reactions between SN and Li metal, suppress the growth of Li dendrites and ameliorate the interfacial stability during the long‐term charging/discharging process, which is favorable to improving cycling stability of SLBs.

Various safety characterization of soft packed LiCoO_2_/PSL53/Li metal batteries are conducted comprehensively. It is clearly seen in Video S1, Figures S31 and S32 (Supporting Information), soft packed LiCoO_2_/PSL53/Li metal batteries still power light‐emitting diode (LED) even after being bent, cut and stroked. This superior safety characteristic can be further evidenced from Video S1 (Supporting Information), soft packed LiCoO_2_/PSL53/Li metal battery can still power LED even the cell has been cut and struck by a hammer continuously. These results demonstrate that PSL53 endows superior safety characteristics for the in situ generated SLBs.

In situ generated PSL53 is also compatible with LiNi_0.6_Co_0.2_Mn_0.2_O_2_ cathode. Rate capability and cycle performance of 4.3 V LiNi_0.6_Co_0.2_Mn_0.2_O_2_/PSL53/Li metal batteries are presented in Figure S34 (Supporting Information). It is obviously to find that the batteries can deliver excellent rate capability (130 mA h g^−1^ at 1 C) and superior cycling stability (capacity retention of 83.5% after 170 cycles) at room temperature, fully demonstrating the universal applicability of this SPE. Above‐mentioned superior comprehensive performance validates PSL53 as an appealing multifunctional SPE for room‐temperature SLBs.

In summary, we have carefully designed a creative strategy that cheap and commercially available TXE as precursor of SPE and LiDFOB as lithium salt and initiator to initiate the in situ polymerization of novel eutectic solvent. This SPE is designed with incomparable advantages including cheap and commercially available raw materials, nonvolatile precursors and free of extra initiators. The as‐assembled 4.3 V LiCoO_2_/Li metal battery delivers superior cycling stability (capacity retention of 88% after 200 cycles) at room temperature. This outstanding battery performance is mainly ascribed to the powerful polymer‐based protective layers formed on LiCoO_2_ and Li simultaneously, which can effectively ameliorate the interfacial compatibility and suppress the continuous growth of lithium dendrites. It is noted that this creative design concept and superior electrochemical performance of this SPE can be extended to other rechargeable room‐temperature solid‐state batteries and enlighten highly‐safe and high‐energy solid‐state batteries.

## Experimental Section

All experimental details are included in the Supporting Information.

## Conflict of Interest

The authors declare no conflict of interest.

## Supporting information

Supporting Information

Supplemental Video 1
