# Supplementary material for: LiDFOB Initiated In Situ Polymerization of Novel Eutectic Solution Enables Room‐Temperature Solid Lithium Metal Batteries
Source: Adv Sci (Weinh). 2020 Nov 3;7(23):2003370. doi: 10.1002/advs.202003370 (PMC7709999; doi:10.1002/advs.202003370)
Supplement: Supplementary file 1 — Supporting Information [file ADVS-7-2003370-s001.pdf]

© 2020 Wiley-VCH GmbH

ADVANCED SCIENCE
Open Access

Supporting Information

for *Adv. Sci.,* DOI: 10.1002/advs.202003370

LiDFOB initiated *in-situ* polymerization of novel eutectic solution enables room-temperature solid lithium metal batteries

Han Wu, Ben Tang, Xiaofan Du, Jianjun Zhang[*]*, Xinrun Yu, Yantao Wang, Jun Ma, Qian Zhou, Jingwen Zhao, Shanmu Dong, Gaojie Xu, Jinning Zhang, Hai Xu*[*]*, Guanglei Cui*[*]*, Liquan Chen*[c]

Supporting Information

LiDFOB initiated *in-situ* polymerization of novel eutectic solution enables room-temperature solid lithium metal batteries

Han Wu[a,b, ‡], Ben Tang[a, ‡], Xiaofan Du[a, ‡], Jianjun Zhang[a,*], Xinrun Yu[a], Yantao Wang[a], Jun Ma[a], Qian Zhou[a], Jingwen Zhao[a], Shanmu Dong[a], Gaojie Xu[a], Jinning Zhang[a], Hai Xu[b,*], Guanglei Cui[a,*], Liquan Chen[c]

[‡]These authors contribute equally to this work.

Section I. The details of experimental section

Preparation of $LiCoO_2$ cathodes and $LiNi_{0.6}Co_{0.2}Mn_{0.2}O_2$ cathodes: $LiCoO_2$ cathodes were fabricated as follows: First, mixing $LiCoO_2$, Super P and polyvinylidene difluoride binder with a weight ratio of 8:1:1. Then, the slurry was coated on an aluminum foil and dried at 60 ^oC for 24 hrs to obtain $LiCoO_2$ cathodes with various areal density. $LiNi_{0.6}Co_{0.2}Mn_{0.2}O_2$ cathode was also fabricated at the same process and the active material loading is about 1.3 mg cm^{-2}.

Preparation of SN-LiDFOB and solid-state polymer electrolytes (PSLs): The raw materials, including LiDFOB (TCI America; 98.0%), succinonitrile (Energy Chemical; 99%) and 1,3,5-Trioxane (Macklin; 99.5%) were stored in the glove box and used without any further purification. SN-LiDFOB was prepared by mixing 4 g SN and 0.365 g LiDFOB at 80 ^oC.

The fabrication of *in-situ* generated solid state polymer electrolytes (PSLs) were as follows: (1) Mixing 2.5 g TXE and the desired amounts of SN (the mass ratios of TXE and SN were 5:1, 5:2, 5:3, 5:4 and 5:5, abbreviated as PSL51, PSL52, PSL53, PSL54 and PSL55, respectively) and then melted at the elevated temperature of 80 ^oC for 10 mins to obtain eutectic solution. (2) 0.365 g LiDFOB was then added into the above-mentioned eutectic solution and stirred to obtain transparent precursor. (3) The precursor was kept at 80 ^oC for a period of time. During this process, LiDFOB will initiate the *in-situ* polymerization of eutectic solution to generate solid-state polymer electrolytes (PSLs).

The fabrication process of *in-situ* generated solid-state lithium metal batteries were as follows: The precursor was injected into lithium metal batteries, where cellulose nonwoven as a rigid supporting and separating material. Finally, lithium metal batteries were kept at 80 ^oC for 1 h to finish the polymerization. The coin cells were filled with 0.09 g electrolyte precursor and the thickness of Li metal is 0.646 mm.

And the parameter of pouch cells is as follows: 0.75 g electrolyte precursor, 0.5 g Li metal and 0.055 g cathode. The energy density of pouch cell is 10.72 Wh kg^{-1} (Calculated based on the total weight of the battery).

Separation and purification of POM from PSLs: POM was separated and purified as follows: PSLs were dispersed with acetonitrile and then the mixed solution was centrifuged at 8000 rpm for 10 mins. Here, acetonitrile was used to dissolve LiDFOB, SN and other soluble components. The above process was repeated five times to separate the white precipitate. The obtained white precipitate was dried at 45 ^{o}C to obtain POM.

Characterizations: POM separated from PSLs is dissolved in 1,1,1,3,3,3-Hexafluoro-2-propanol-d2 for ^{1}H NMR and ^{13}C NMR analysis on a Bruker AVANCE III 600 MHz with tetramethylsilane as internal reference. GPC measurements were performed by dissolving the POM in 1,1,1,3,3,3-Hexafluoro-2-propanol and eluting the solutions in room temperature GPC (Waters) equipped with triple detection capability for absolute polymer molecular weight determination. Infrared spectra measurements were conducted on a Fourier transform infrared spectrometer (Bruker VERTEX 70). Ionic conductivity was performed using a BioLogic VSP-300. The cycled cathode and anode are washed by 1,2-Dimethoxyethane (DME) for three times and stored in the 20 mL DME over night to remove the attached SPE. Cross-sections SEM images of the Li metal anode cycled with SN-LiDFOB was observed by SEM after ion milling (IM4000PLUS, HITACHI Co., Ltd, Japan) using an argon ion beam. The surface morphology of $LiCoO_2$ cathodes and lithium metal anodes were characterized by using TEM (80 kV). DSC was tested using a Q1000 Modulated Differential Scanning Calorimeter (TA Instruments). AnSSX-100 XPS spectrometer was used to study the surface component of $LiCoO_2$ cathodes and Li metal anodes. Rate capability and cycle performance of coin-type cells (CR2032) using SN-LiODFB and PSL53 were conducted on LAND testing system (Wuhan LAND electronics Co., Ltd.) at room temperature and -10 ^{o}C, respectively.

Calculation methods: All quantum chemical calculations were performed by applying the density functional theroy (DFT) method with the B3LYP level and 6-311+G (d, p) basis set using Gaussian 09 program package. The structural optimization was determined by minimizing the energy without imposing molecular symmetry constraints. The interaction energies of the POM-SN were defined as the

interaction between different molecule fragments. The interaction energy E_1 was calculated according to equation (1), the expression as follows:

$$E_1 = E_{total} - nE_Y \qquad (1)$$

where E_{total} is the structure total energy, E_Y is the energy of different molecule fragments (Y = POM, TXE, SN), and n is the number of corresponding molecule fragments according to the different structure configurations.

The first-principles calculation based on the density functional theory (DFT) method performed in this study are conducted out with the Vienna ab initio simulation package (VASP)[1] within the projector augmented-wave (PAW) approach.[2] The generalized gradient approximation is adopted in the parameterization of Perdew, Burke, and Ernzerhof (PBE)[3] to describe the exchange-correlation functional. For our simulated structures, the kinetic energy cutoffs were set at 500 eV for the plane wave basis set and the calculation were non-spin-polarized.[4] The Brillouin zone integrations used Monkhorst-Pack grids of 2×2×1 mesh. The structure relaxation was considered completed with the conjugate gradient method when a force convergence criterion was less than 0.02 eV $Å^{-1}$ and total energy convergence within 1×10^{-5} eV per unit cell. The cell optimization of the Li was calculated within the 2×2×2 supercell consisting of 16 atoms. The Li- (110) slab consists of 4 layers and an xy-plane of dimensions 12.0 Å x 12.0 Å with 20 Å of vacuum in the z-direction. In order to calculate the adsorption energies E_2 of POM, SN and $DFOB^-$ on Li- (110) slab, we use the following formula (2):

$$E_2 = E_{tot} - E_{sheet} - E_X \qquad (2)$$

where E_{tot}, E_{sheet} and E_X represents the total energy of the system, energy of Li- (110) slab and energy of POM, SN and $DFOB^-$.

MD simulations: To understand the effect of the second electrolyte solvent POM on the microscopic structures of SN/LiDFOB mixtures, one ternary POM/SN/LiDFOB mixture was investigated by Molecular dynamic (MD) simulations. First, the initial structure of the mixture system was set up by randomly placing the number of LiDFOB, POM and SN molecules to construct the bulk systems based on the experimental densities and the molar ration (concentration). The size of the simulation system is

48.8×48.8×48.8 Å^3, the total number of atoms is 9800. The molar ratios between the solvents and salts used in our simulations was 3:30:20 for LiDFOB:POM:SN. The simulation cells contained 30 LiDFOB, 300 POM and 200 SN. Subsequently, all mixture systems were equilibrated by NPT (Isothermal isobaric ensemble) MD simulations for 5ns at 353K and atmospheric pressure, followed by NVT **(***canonical ensemble***) MD simulations for 10ns** at 353K **with a 1 fs time step.** All MD simulations were performed using the Forcite code with Universal force field[5]. The temperature was controlled by a Nose-Hoover Langevin (NHL) thermostat and the pressure was controlled by a Berendsen barostat[6]. The Ewald scheme[7] and atom-based cutoff method (*i.e.*, a radius of 15.5 Å) were applied to treat electrostatic and van der Waals (vdW) interactions, respectively. All the partial atomic charges were defined using the Universal force field.

Section II. Supporting figures and relevant discussion:

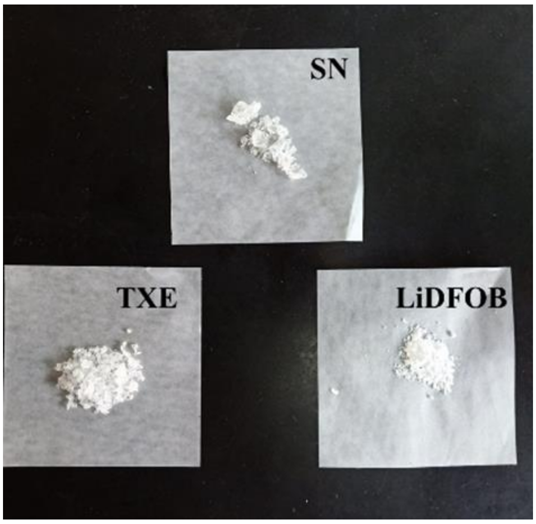

Figure S1. Digital images of SN, TXE and LiDFOB at room temperature.

Figure S1 displays the physical state of three raw materials (SN, TXE and LiDFOB) of solid state polymer electrolytes (PSLs) at room temperature. Obviously, all raw materials are totally solid and non-volatile at room temperature, which demonstrates our reasonal design that there is no volatile or flammable precursor in the following solid-state polymer electrolytes (PSLs).

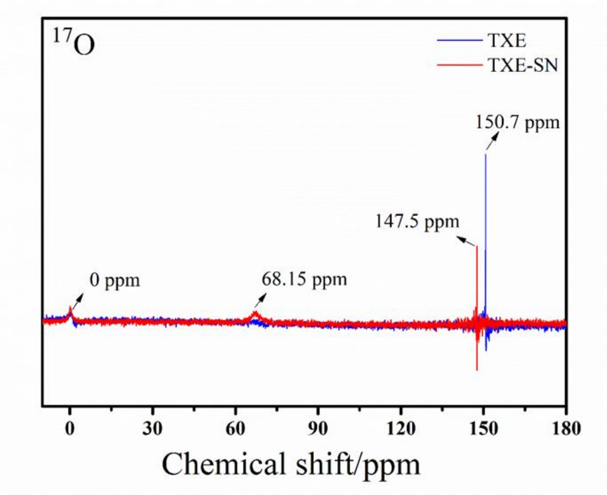

Figure S2. ^{17}O NMR spectra of TXE and TXE-SN eutectic solution.

As shown in **Figure S2**, the peak at 0 ppm belongs to H_2O, and the peak at 150.7 ppm is characteristic peak of TXE. After mixing with SN, TXE's chemical shift changes from 150.7 ppm to 147.5 ppm, and a new peak forms at 68.15 ppm, indicating that TXE have interacted with SN.

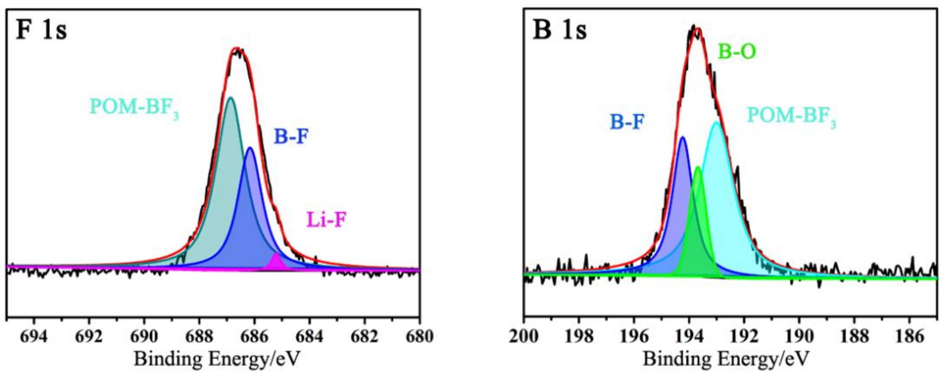

Figure S3. XPS spectra (F 1s and B 1s) of PSL53.

In F 1s spectra of PSL53, an obvious signal (686.8 eV) belonging to BF_3 attached to polymer chain ($POM-BF_3$) and weak signal belonging to LiF (685 eV) were spotted. What is more, in B 1s spectra, the corresponding signal (192.7 eV) of $POM-BF_3$ also indicates the existence of BF_3.

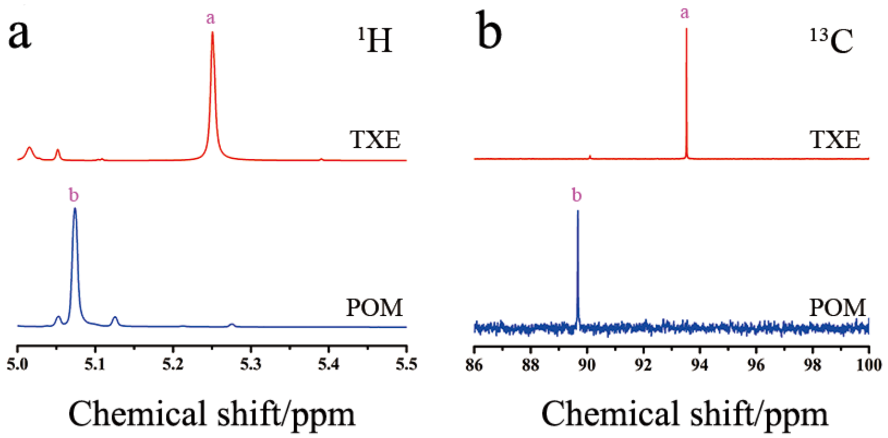

Figure S4. NMR spectra of TXE and POM. (a) Hydrogen and (b) carbon NMR spectra of TXE and POM.

As displayed in **Figure S4a** and **Figure S4b**, new hydrogen peak (5.07 ppm) and carbon peak (89.7 ppm) are observed in the NMR spectra of POM after polymerization, which are in well accordance with the structure of POM, indicating that TXE has successfully polymerized to POM.

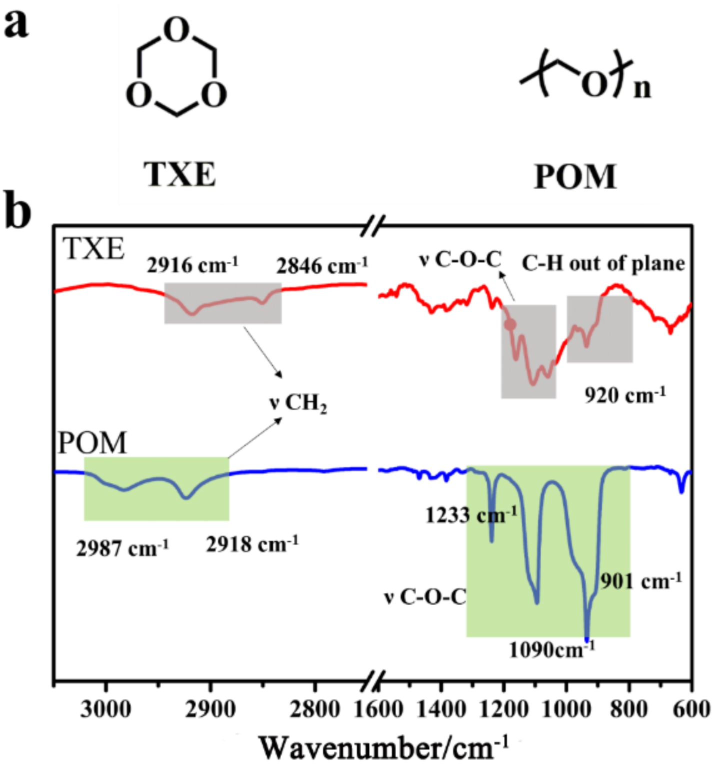

Figure S5. Structure characterization by FTIR. (a) Chemical structure of TXE and POM. (b) FTIR spectra of pristine TXE, and POM separated from PSL53.

As shown in **Figure S5**, after the polymerization, the doublet absorption peaks attribute to stretching vibration mode of O-CH_2-O group (TXE) shifts from 2846 and 2916 cm^{-1} to 2918 cm^{-1} and 2987 cm^{-1}, respectively. In addition, the missing of C-H out of plane vibration at 920 cm^{-1} and the emerging of absorption peaks at 1233 cm^{-1}, 1090 cm^{-1} and 901 cm^{-1} resulting from the CH_2-O-CH_2 stretching mode also validates that TXE has been successfully polymerized to POM.[8]

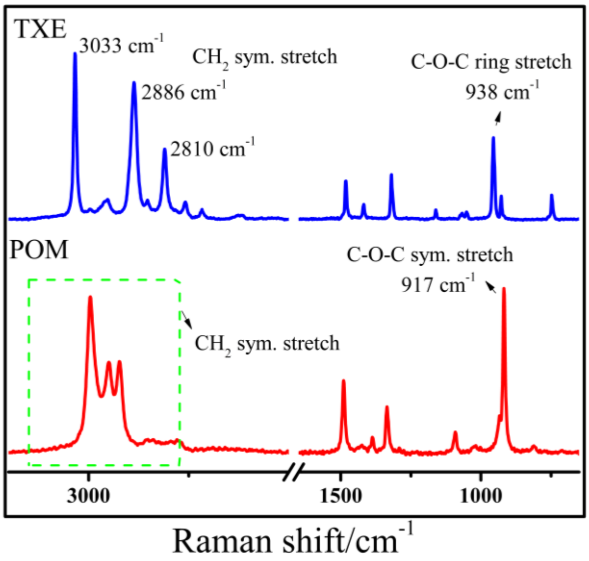

Figure S6. Structure characterization by Raman. Raman spectra of pristine TXE, and POM separated from PSL53.

The result of Raman characterization is consistent with the finding of FTIR spectra. After the polymerization, the signal (938 cm^{-1}) belonging to C-O-C ring stretching is missing. In addition, new peak (917 cm^{-1}) associated with C-O-C symmetric stretching appears. And peaks belonging to CH_2 symmetric stretching have shift from 2810 cm^{-1}, 2886 cm^{-1}, 3033 cm^{-1} to 2922 cm^{-1}, 2949 cm^{-1}, 2995 cm^{-1} respectively, suggesting the successful polymerization of TXE.[9]

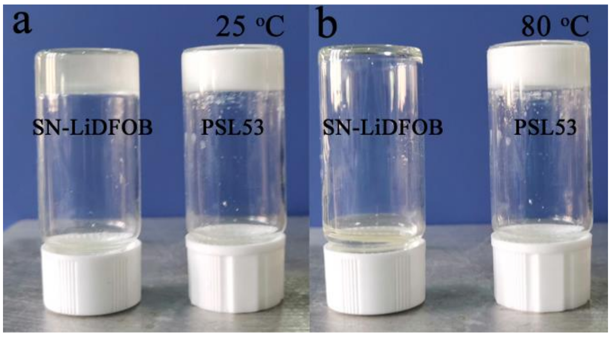

Figure S7. Digital images of SN-LiDFOB and PSL53 at (a) room temperature and (b) 80 ^{o}C.

As shown in **Figure S7a**, SN-LiDFOB (the mass ratio of SN and LiDFOB is 4:0.365) is immobile at room temperature. While at the elevated temperature of 80 ^{o}C, SN-LiDFOB electrolyte turns into a flowable liquid solution (**Figure S7b**). In a sharp contrast, PSL53 is a white solid state polymer electrolyte at room temperature. More importantly, PSL53 remains solid even at the elevated temperature of 80 ^{o}C.

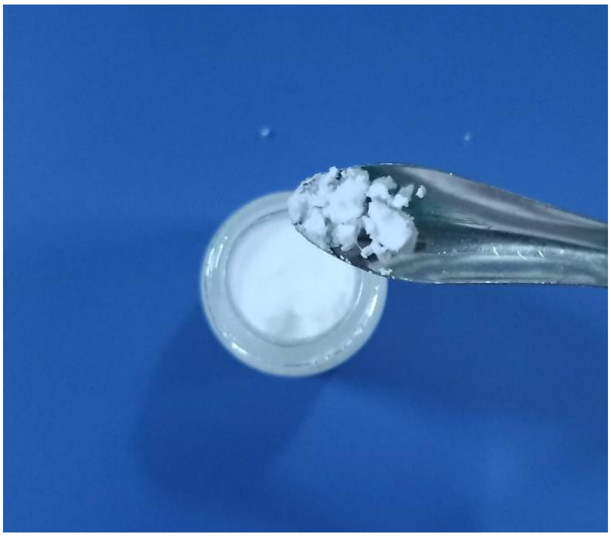

Figure S8. Digital photo of PSL53

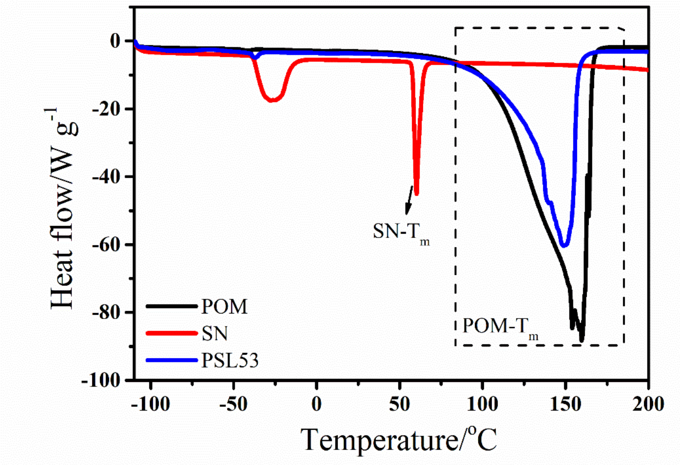

Figure S9. DSC curves of SN, POM and PSL53.

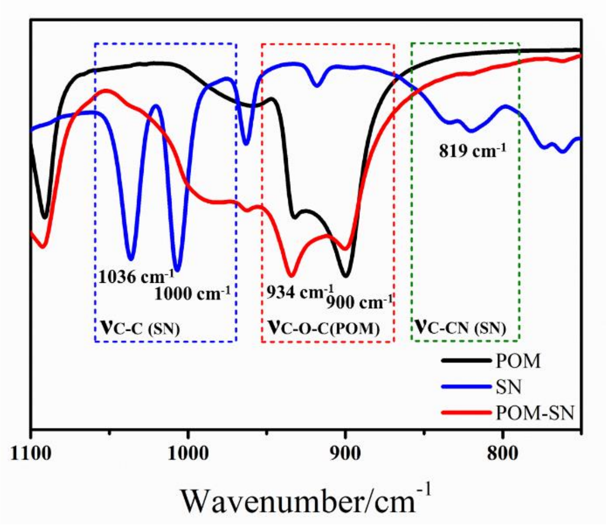

Figure S10. FTIR spectra of POM, SN and POM-SN.

As shown in **Figure S10**, after mixed with SN, the peak belonging to the stretching vibration mode of C-O-C (POM) shifts from 900 cm^{-1} to 934 cm^{-1}, manifesting the strong interaction between POM and SN.

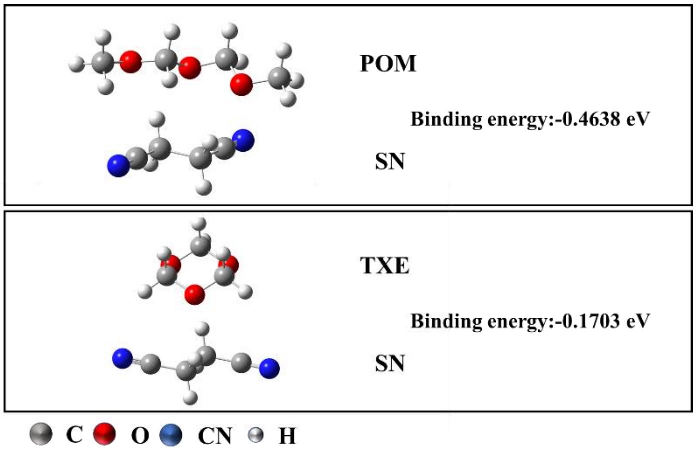

Figure S11. Binding energy between POM or TXE and SN.

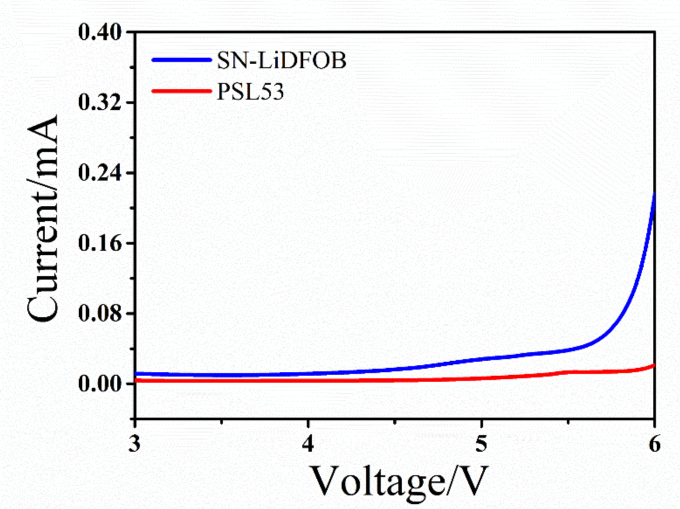

Figure S12. Linear sweep voltammetry (LSV) curve of Stainless steel||Li cells in SN-LiDFOB and PSL53.

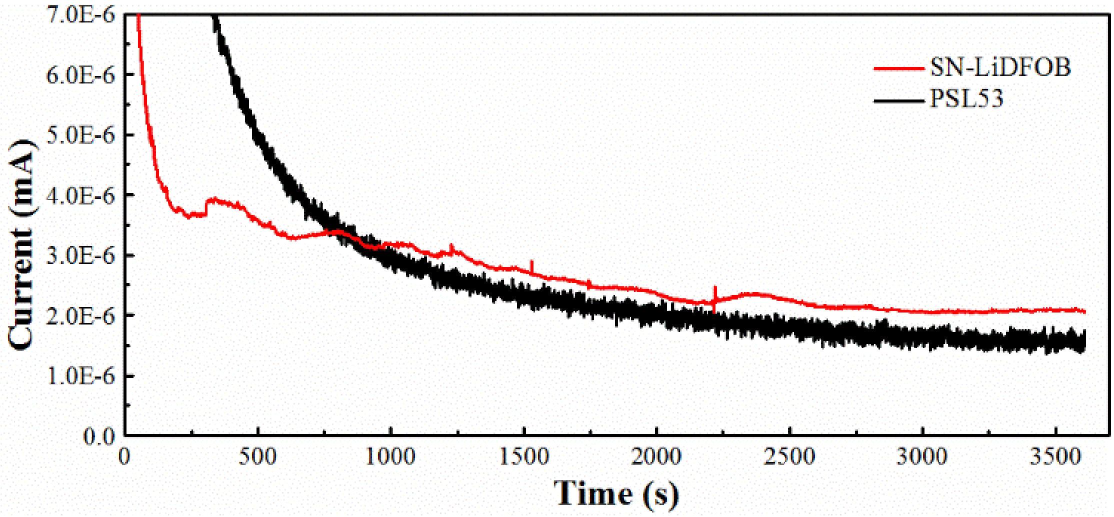

Figure S13. Current-time curves of the Stainless steel/electrolyte/Stainless steel symmetric cells under DC polarization at 100 mV.

The room temperature electronic conductivity is measured by the current-time curves of the stainless steel/electrolyte/stainless steel cells under DC polarization at 100 mV (**Figure S13**). The current first decreased and then keep steady, at the steady state, the current is contributed only by the migrating electrons in electrolyte due to the ion-blocking stainless steel electrode is used. According to the following formulation, the electronic conductivity of PSL53 is calculated as 2.7×10^{-8} S cm^{-1}, and the electronic conductivity of SN-LiDFOB is 3.6×10^{-8} S cm^{-1}, which is low enough for lithium batteries[10].

$$\sigma = \frac{L}{RS}$$

σ is the electronic conductivity, L is the thickness of the electrolyte, S is the surface area of stainless

steel electrode, and R is the resistance of electrode, which can be calculated by Ohm's Law ($R=U/I_s$, where U is the polarization voltage, I_s is the current of steady state)

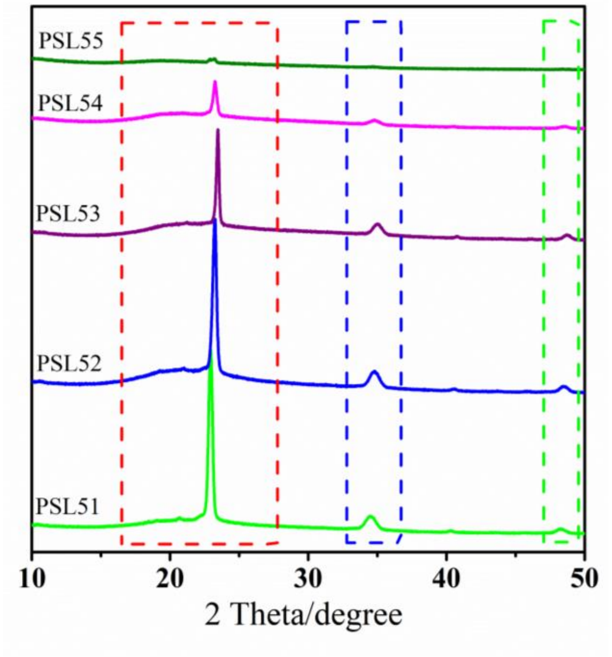

Figure S14. XRD patterns of electrolytes with various concentrations of SN.

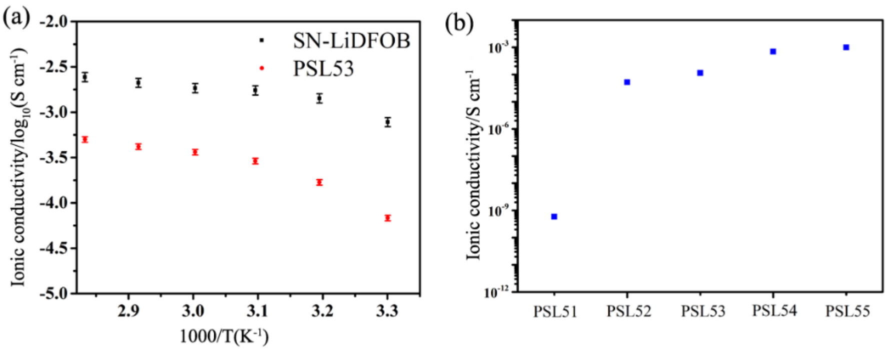

Figure S15. (a) The ionic conductivity comparison of SN-LiDFOB and PSL53. (b) Room-temperature ionic conductivities of electrolytes with various concentrations of SN.

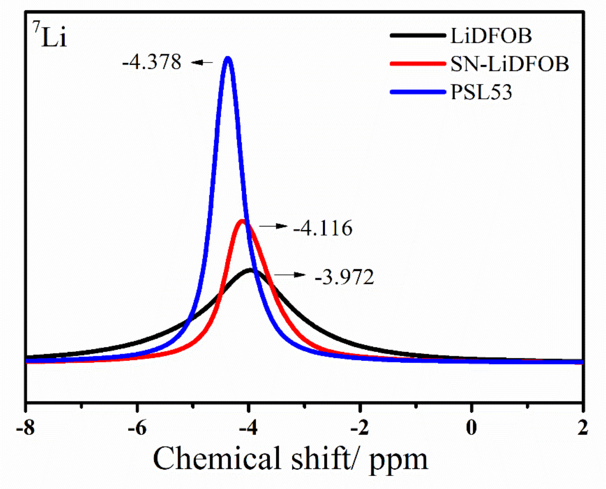

Figure S16. ^{7}Li MAS NMR of LiDFOB, SN-LiDFOB and PSL53.

The analysis of ^{7}Li solid state NMR spectra allows the identification of mobile and immobile cations, since the line broadening of the signals resulting from homo- and heteronuclear magnetic dipolar couplings will be averaged in the presence of motional processes. As depicted in **Figure S16**, these changes of chemical shift indicate that both SN and POM interact with Li cations. As is well known, for ^{7}Li solid state NMR spectra, the linewidth of peak is inversely proportional to the mobility of cations.[11] The linewidth of PSL53 is 3.4 ppm, which is slightly higher than that (3.3 ppm) of SN-LiDFOB, manifesting that Li cations in PSL53 are approachable as that of SN-LiDFOB. This result is in accordance with the trend of ionic conductivity **(Figure S16a).**

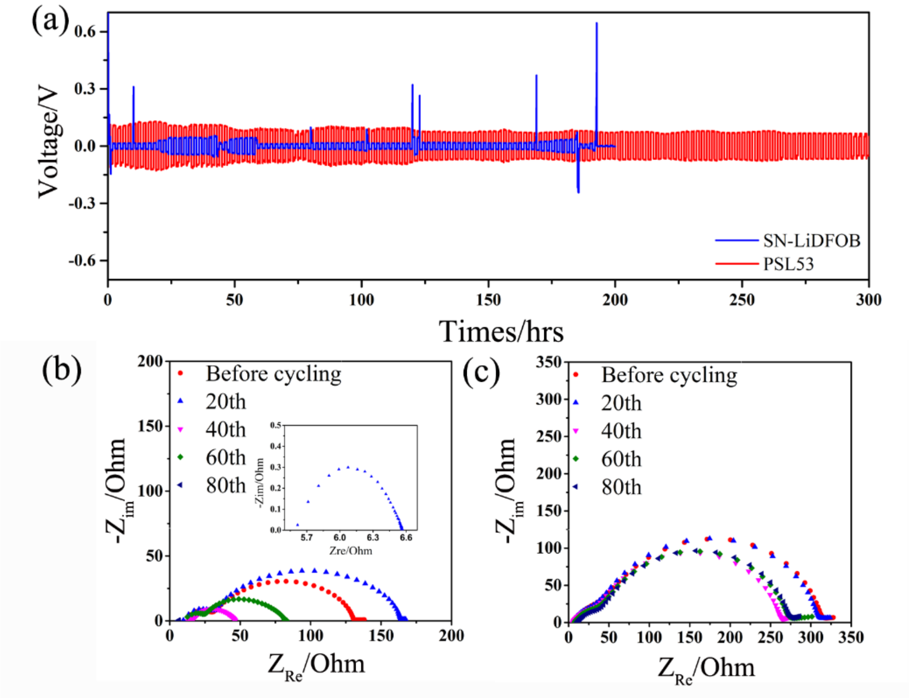

Figure S17. (a) Galvanostatic cycling curves of Li/Li symmetrical cells using SN-LiDFOB and PSL53. (b) EISs of Li/SN-LiDFOB/Li cell after varied cycles. (The inset in **Figure S17b** is the enlarged EISs of Li/SN-LiDFOB/Li cell after 80 cycles) (c) EISs of Li/PSL53/Li cell after varied cycles.

It is now well recognized that low Coulombic efficiency and safety risks is usually induced by uncontrollable growth of lithium dendrites. Thus, Li/Li symmetric cells were assembled and used to investigate the interfacial compatibility of electrolytes (SN-LiDFOB and PSL53) with Li metal anode. For the Li/SN-LiDFOB/Li cell, the potential dramatically fluctuates due to the non-uniform Li plating/stripping behavior and parasitic side-reactions between SN and Li metal. Meanwhile, the potential suddenly drops after 80 hrs cycling, indicating short-circuit mainly caused by the continuous growth of Li dendrites (**Figure S17a**). Moreover, the EIS results (**Figure S17b**) of SN-LiDFOB based Li/Li cell also collaborate this finding. In a sharp contrast, the PSL53 based Li/Li symmetrical cell exhibits much more stable Li plating/stripping profiles than that of SN-LiDFOB for over 300 hrs. Furthermore, the EIS results (**Figure S17c**) of the Li/PSL53/Li symmetrical cell have minor change as cycling. These results demonstrate that PSL53 shows superior interfacial compatibility with Li metal anode. It corrugates that the continuous side-reactions between SN and Li metal is significantly suppressed, which is advantageous to improve cycle performance of SLB.

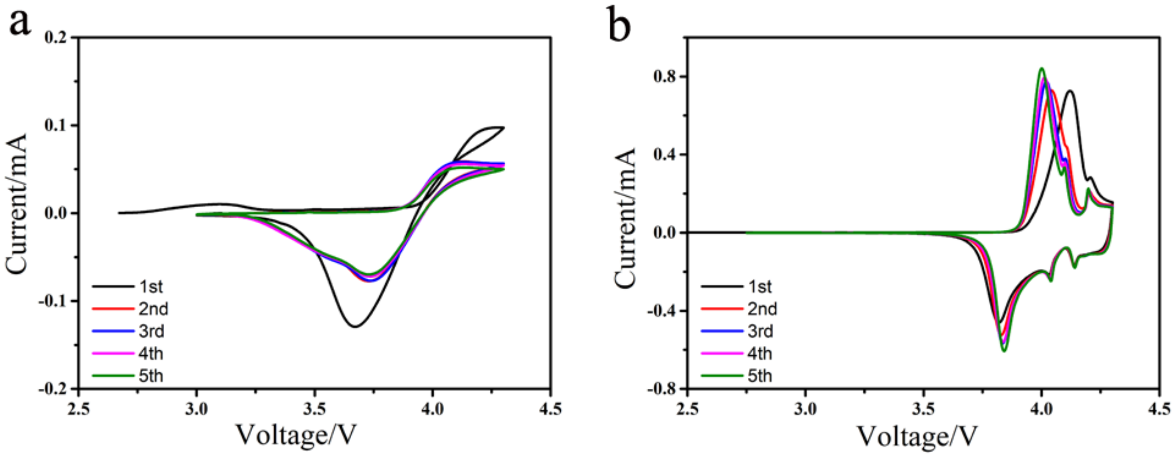

Figure S18. CV curves of $LiCoO_2$/Li metal batteries with the electrolytes of (a) SN-LiDFOB and (b) PSL53.

CV curve can provide critical information on the electrochemical stabilty of electrolytes. As depicted in

Figure S18b, CV curve of $LiCoO_2$/PSL53/Li metal battery is much more symmetrical and rational than that of $LiCoO_2$/SN-LiDFOB/Li metal battery (**Figure S18a**), which also corroborats the interfacial compatibility and stability between PSL53 and varied electrodes. The inferior electrochemical performance of $LiCoO_2$/SN-LiDFOB/Li metal battery is mainly atrributed to the poor interfacial stability deriving from parasitic side-reactions between SN and Li metal.

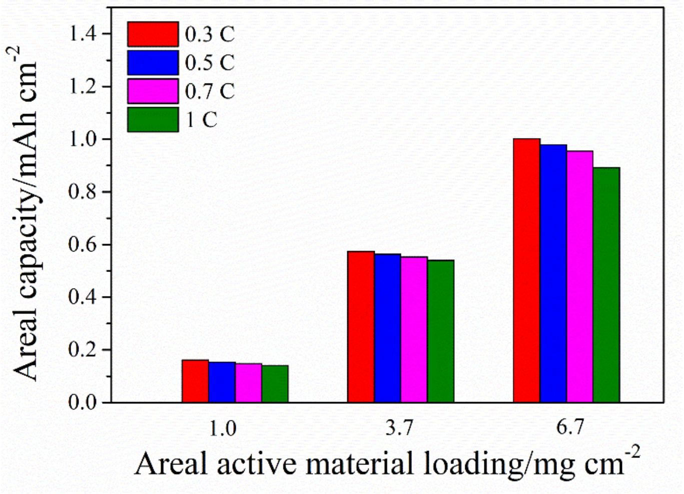

Figure S19. Rate capability of 4.3 V $LiCoO_2$/PSL53/Li metal batteries at varied areal active material loadings.

As shown in **Figure S19**, 4.3 V $LiCoO_2$/PSL53/Li metal batteries displays excellent rate capability, which are much better than those of most previously reported works.[8-11] Even at a high current density of 1 C, only minor areal capacity fading can be spotted in varied areal active material loadings (1.0, 3.7 and 6.7 mg cm^{-2}), manifesting the excellent rate capability of solid-state 4.3 V $LiCoO_2$/PSL53/Li metal batteries.

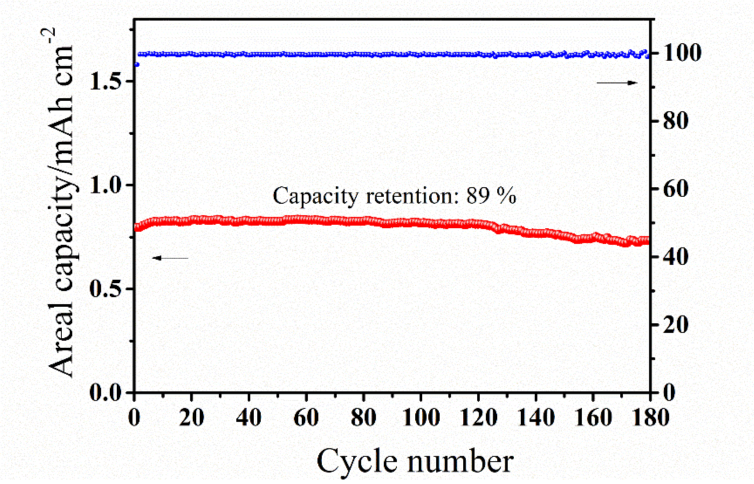

Figure S20. Cycle performance of 4.3 V $LiCoO_2$/PSL53/Li metal battery at the rate of 0.1 C at the active material mass loading of 5.8 mg/cm^{-2}.

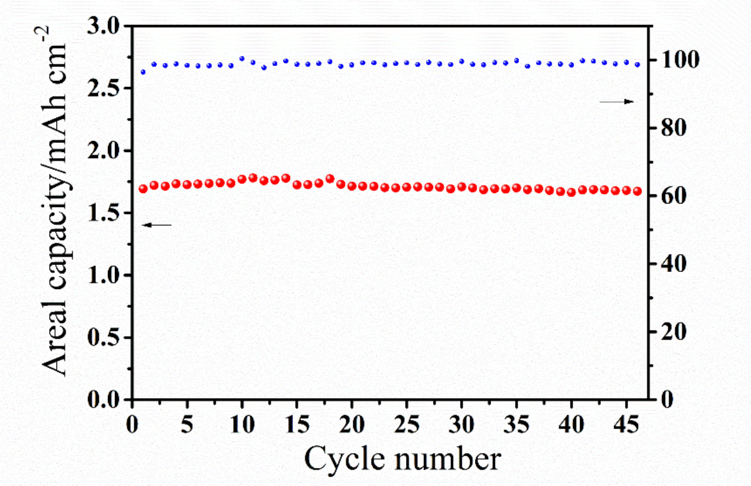

Figure S21. Cycle performance of 4.3 V $LiCoO_2$/PSL53/Li metal battery at the rate of 0.1 C at the active material mass loading of 11.5 mg/cm^{-2}.

As shown in **Figure S21**, even at a high active material mass loading of 11.5 mg cm^{-2}, 4.3 V $LiCoO_2$/PSL53/Li metal battery can still deliver a favorable areal capacity of 1.72 mA h cm^{-2} even after 45 cycles (initial areal capacity is 1.75 mA h cm^{-2}), indicating excellent cycling stability of this solid-state lithium metal batteries.

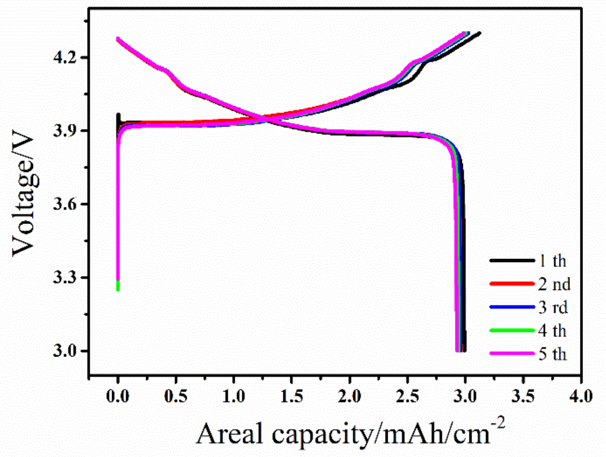

Figure S22. Charge/discharge profiles of 4.3 V $LiCoO_2$/PSL53/Li metal battery at the rate of 0.05 C at room temperature (mass loading: 18.58 mg cm^{-2}).

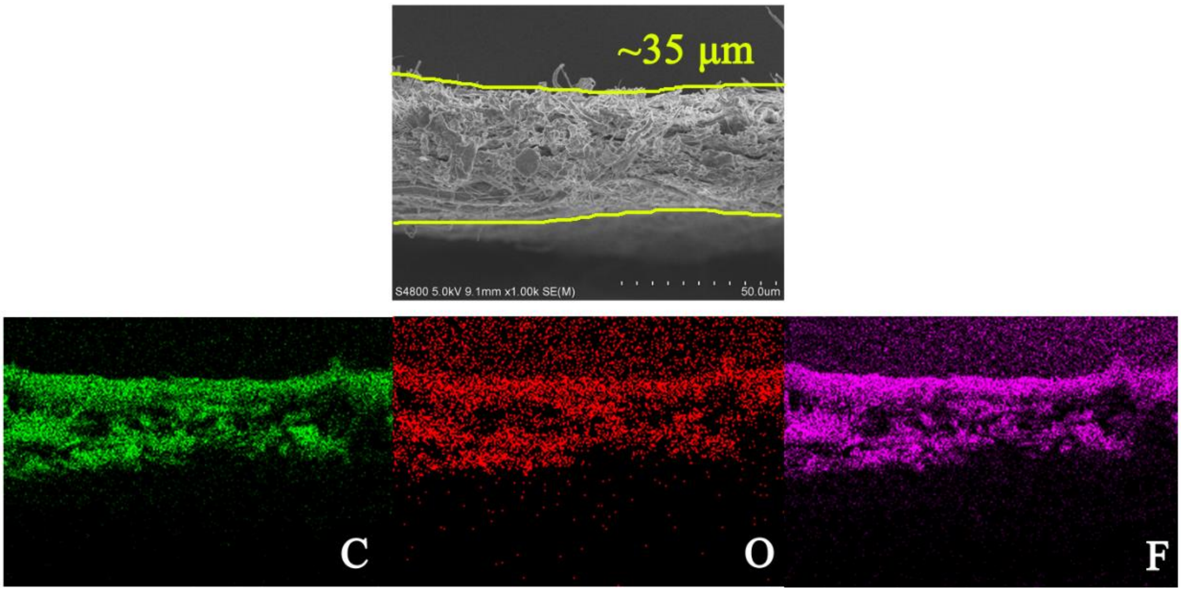

Figure S23. Typical SEM images and EDS of *in-situ* formed PSL53.

According to the SEM picuture dismatled from pre-prepared battery using PSL53, we have concluded that the average thickness of the electrolyte is 35 μm.

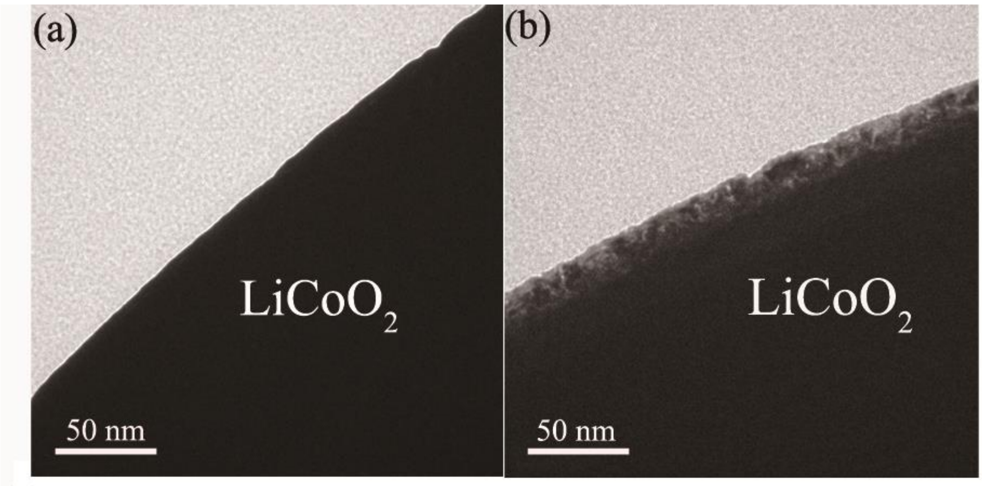

Figure S24. Typical TEM images of $LiCoO_2$ cathdoe cycled with (a) SN-LiDFOB and (b) PSL53.

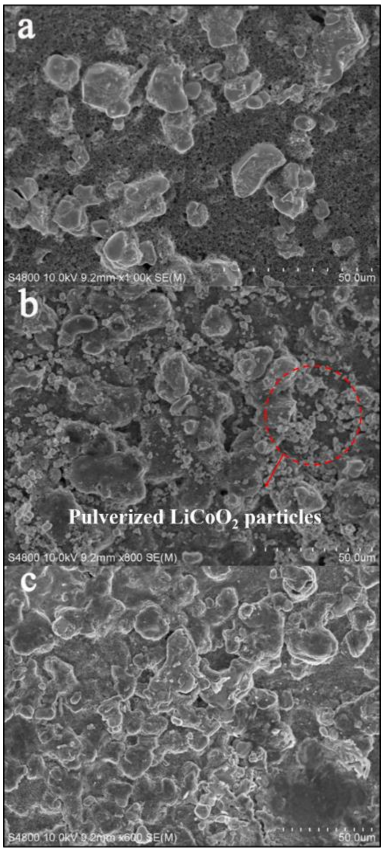

Figure S25. Typical SEM images of $LiCoO_2$ cathodes. (a) Pristine $LiCoO_2$ cathode. Cycled $LiCoO_2$ cathodes disassembled from $LiCoO_2$/Li metal batteries using (b) SN-LiDFOB and (c) PSL53.

More uniform and homogeneous morphology is found on cycled $LiCoO_2$ cathode disassembled from $LiCoO_2$/PSL53/Li metal battery, which is in sharp contrast with that of $LiCoO_2$/SN-LiDFOB/Li metal battery.

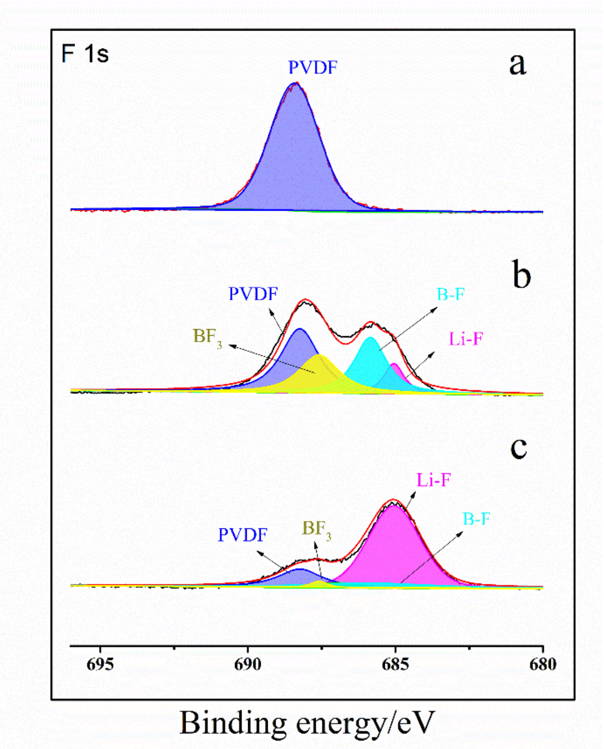

Figure S26. XPS spectra (F 1s) of $LiCoO_2$ cathodes. (a) Uncycled $LiCoO_2$ cathode. Cycled $LiCoO_2$ cathode disassembled from $LiCoO_2$/Li metal batteries with (b) SN-LiDFOB and (c) PSL53.

For the cycled $LiCoO_2$ cathode with SN-LiDFOB, weak signal belonging to Li-F is detected. In contrast, extraordinarily strong signal (685 eV) belonging to Li-F is spotted on the surface of cycled $LiCoO_2$ cathode with PSL53, indicating PSL53 favors to generate the LiF-rich passivation layer on the surface of $LiCoO_2$.

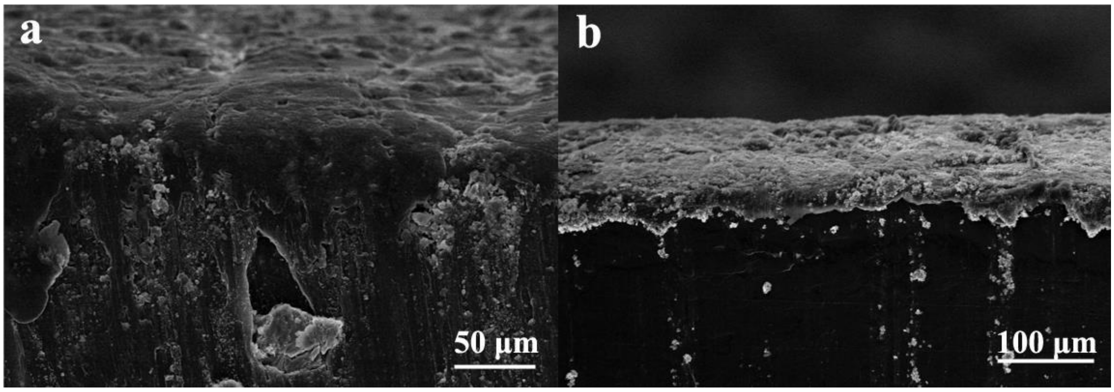

Figure S27. Typical cross-section SEM images of cycled Li metal anodes disassembled from $LiCoO_2$/Li metal batteries with (a) SN-LiDFOB and (b) PSL53.

As clearly shown in the **Figure S27a**, there are more erosions on the cross section of cycled Li metal anodes disassembled from $LiCoO_2$/SN-LiDFOB/Li metal battery, which is different from that of cycled Li metal anodes disassembled from $LiCoO_2$/PSL53/Li metal battery (**Figure S27b**).

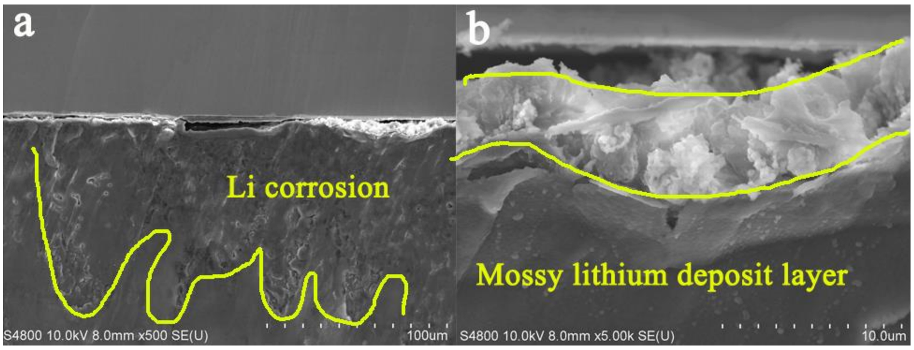

Figure S28. Cross-sections SEM image of the Li metal anode cycled with SN-LiDFOB (prepared by ion milling).

As shown in **Figure S28**, even the sample was prepared by ion milling using an argon ion beam, there are obvious Li corrosion which are detected in the cross-section images. What is more, Mossy lithium deposit layer is also found in the magnified images.

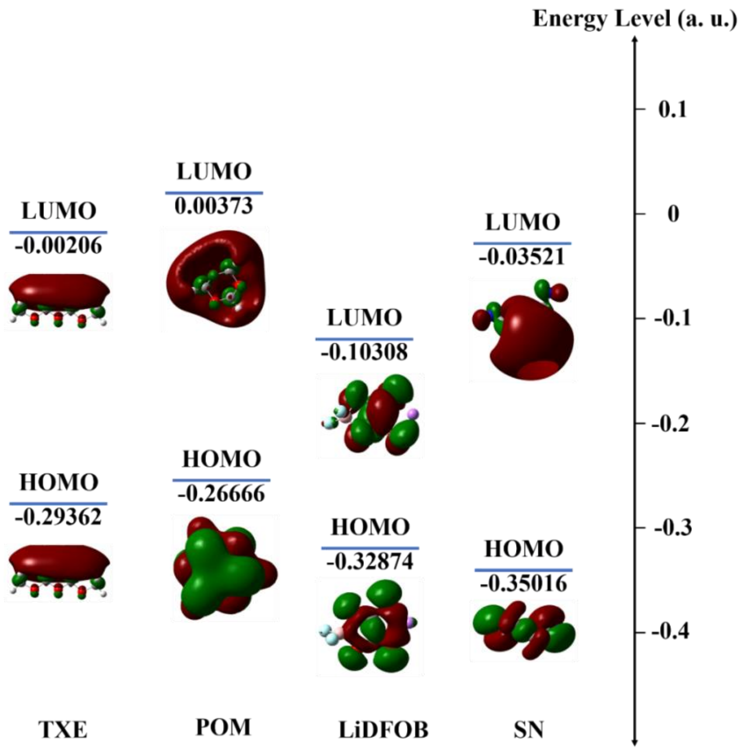

Figure S29. HOMO and LUMO energy level comparison of TXE, POM, LiDFOB and SN along with the correlated Energy-Level diagram.

In order to gain further insight of interfacial stability between PSL53 and lithium metal, theoretical calculations based on density functional theory (DFT) are used to evaluate its thermodynamic stability. As shown in **Figure S29,** the obtained LUMO energy of POM and TXE were 0.00373 a.u. and -0.00206 a.u., respectively. It is common knowledge that the higher LUMO energy indicates that it is more stable and compatible with Li metal anode. Therefore, it can be concluded that POM and TXE is stable with Li metal anode, indicating a superior interfacial compatibility of POM and TXE with Li metal anode.

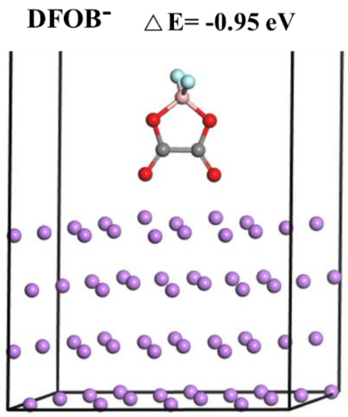

Figure S30. The density functional theory (DFT) calculations on the interface interaction energy between the molecules ($DFOB^-$) and Li-110 slab.

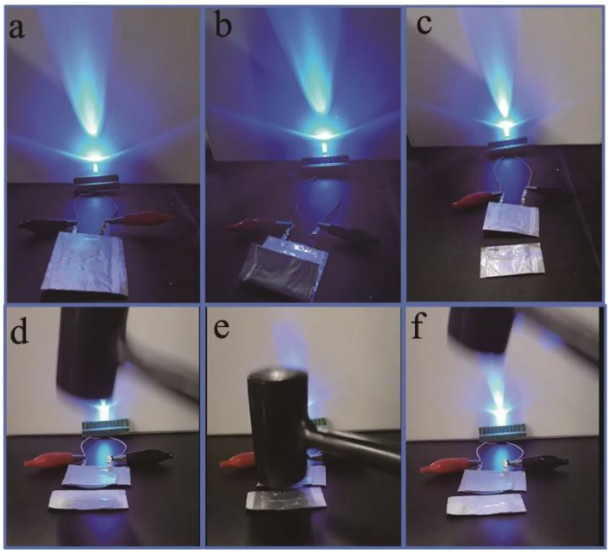

Figure S31. Safety characterization of soft packed $LiCoO_2$/PSL53/Li metal batteries. Digital images of blue light-emitting diode (LED) powered by soft packed $LiCoO_2$/PSL53/Li metal batteries at (a) unbending state, (b) bending state (c) cutting state. (d-f) the being cut battery stroked by a hammer.

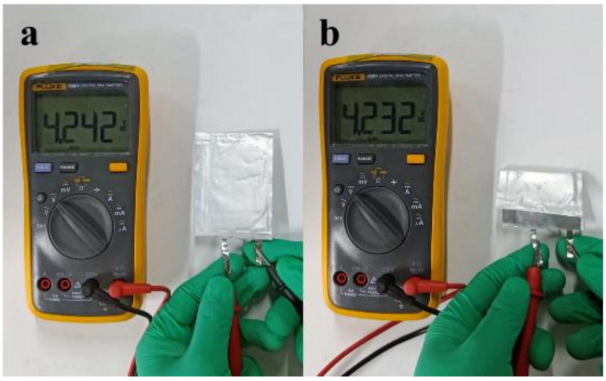

Figure S32. Voltage comparison of soft packed $LiCoO_2$/PSL53/Li metal battery at (a) unbending and (b) bending state.

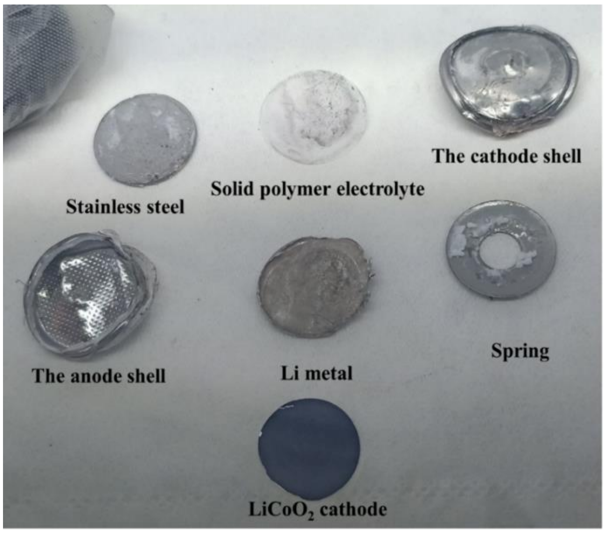

Figure S33. Digital images of key components disassembled from 4.3 V $LiCoO_2$/PSL53/Li metal battery.

Obviously, there is no liquid remainings in $LiCoO_2$/PSL53/Li metal battery, further proving that our PSL53 electrolyte is really solid-state polymer electrolyte.

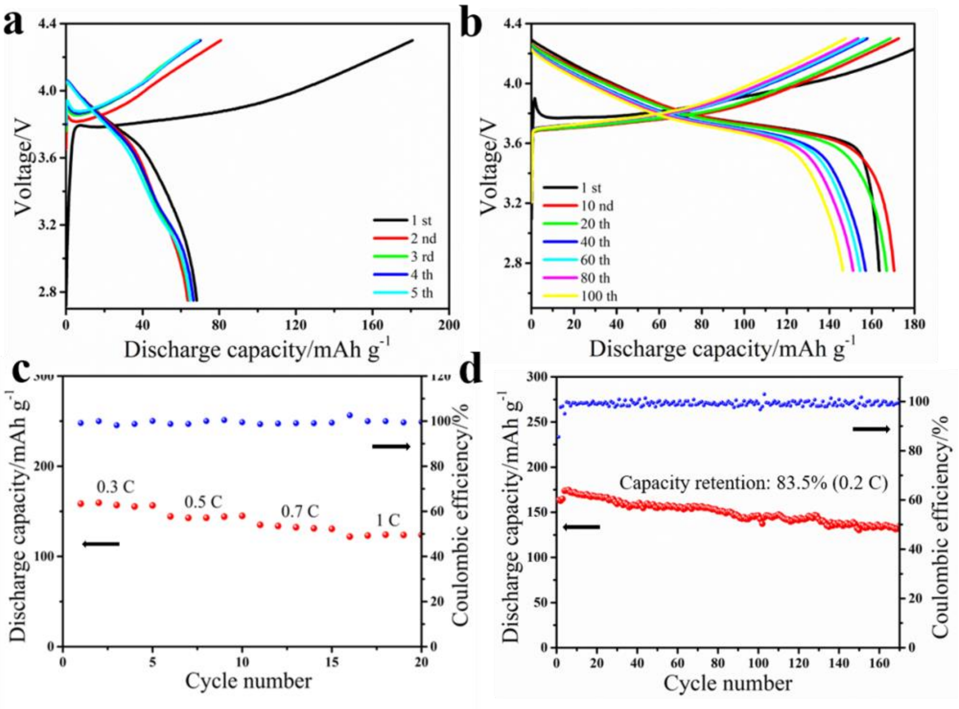

Figure 34. Battery performances of 4.3 V $LiNi_{0.6}Co_{0.2}Mn_{0.2}O_2$/Li metal batteries. (a) Charge/discharge profiles of battery cycled with SN-LiDFOB at the rate of 0.2 C at room temperature. (b) Charge/discharge profiles of 4.3 V battery cycled with PSL53 at the rate of 0.2 C at room temperature. (c) Rate capability and (d) cycling performance of batteries at room temperature.

Table S1 GPC results and the corresponding polymerization time of PSLs with different contents of SN at 80 ^{o}C.

Samples	M_n (Daltons)	M_w (Daltons)	Polydispersity (M_w/M_n)
PSL51	8999	15380	1.709
PSL52	10100	12680	1.256
PSL53	5083	5388	1.060
PSL54	2348	2487	1.059
PSL55	1232	2090	1.696

As listed in **Table S1**, M_n of POM generally decrease with the increasement of SN content, which is precisely the relationship one would expect if SN functioned as an inhibitor for the in situ ring-opening polymerization reaction. Specifically, the higher content of SN retards the reaction rate, causing polymer chain to grow more slowly, which would lower polymer molecular weight. In addition, POM

exhibits relatively narrow polydispersity (1.060) and suitable molecular weight (M_n=5083) when the mass ratio of TXE:SN is 5:3.

To achieve the optimal SPE, we comprehensively investigated the effect of SN content on polymerization time, molecular weight and polydispersity of POM. As depicted **Table. S1**, it is clearly that high content of SN can prolong polymerization time and decrease molecular weight (*Mn*). In addition, PSL53 exhibits suitable molecular weight (*Mn*=5083) and relatively narrow polydispersity (1.060). Hence, we finally choose PSL53 as typical SPE for the further investigation owing to its favorable polymerization time, suitable molecular weight and narrow polydispersity of POM.

Table S2. Performance comparison of our SPE with previously reported *in-situ* generated SPEs.

SPEs	Precursors (their physical state at room temperature)	Initiators	Cycle performance	References
Poly(vinyl carbonate)/LiDFOB	Vinylene carbonate (Liquid)<br>LiDFOB (Solid)	AIBN	84.2% after 150 cycles at 50 ^oC<br>(2.5 V-4.3 V, $LiCoO_2$/Li, 0.1 C)<br>(1.5 mg cm^{-2})	[12]
Poly(dioxolane)/LiTFSI	1,3-dioxolane (Liquid)<br>LiTFSI (Solid)	Aluminium triflate	~73% after 700 cycles at 25 ^oC<br>(2.5 V-4 V, $LiFeO_4$/Li, 1 C)<br>(5 mg cm^{-2})<br>~75% after 200 cycles at 25 ^oC<br>(3 V-4.2 V, NCM_{622}/Li, 0.1 C)	[13]
Poly(tetrahydrofuran)/$LiClO_4$	Tetrahydrofuran (Liquid)<br>$LiClO_4$ (Solid)	Boron trifluoride	91.3% after 100 cycles at 60 ^oC<br>(2.5 V-4 V, $LiFeO_4$/Li, 0.1 C)	[14]
Poly(dioxolane)/LiTFSI	1,3-dioxolane (Liquid)<br>Diallyl disulfide (Solid)<br>LiTFSI (Solid)	$LiPF_6$	80% after 400 cycles at 30 ^oC<br>(2.5 V-4 V, $LiFeO_4$/Li, 3 C)<br>(3 mg cm^{-2})	[15]
Poly(formaldehyde) /SN/ LiDFOB	1,3,5-trioxane (Solid)<br>Succinonitrile (Solid)	LiDFOB	88% after 200 cycles at room temperature<br>(3 V-4.3 V, $LiCoO_2$/Li, 0.3 C) (1.3 mg cm^{-2})<br>97% after 45 cycles at room temperature<br>(3 V-4.3 V, $LiCoO_2$/Li, 0.1 C) (11.5 mg cm^{-2})<br>83.5% after 170 cycles at room temperature (2.75 V-4.3 V, NCM_{622}/Li, 0.2 C)<br>(1.3 mg cm^{-2})	Our work

References

[1] G. Kresse, J. Furthmüller, *Phys. Rev. B* 1996, 54, 11169.

[2] P. E. Blöchl, *Phys. Rev. B* 1994, 50, 17953.

[3] J. Perdew, K. Burke, M. Ernzerhof, *Phys. Rev. Lett.* 1998, 80, 891.

[4] F. Du, X. Ren, J. Yang, J. Liu, W. Zhang, *J. Phys. Chem. C* 2014, 118, 10590.

[5] a) H. J. Sun, *J. Phys. Chem. B* 1998, 102, 7338; b) H. J. C. Berendsen, J. P. M. Postma, W. F. Van Gunsteren, A. Dinola, J. R. Haak, *J. Chem. Phys.* 1998, 81, 3684.

[6] A. A. Samoletov, C. P. Dettmann, M. A. Chaplain, *J. Stat. Phys.* 2007, 128, 1321.

[7] a) P. P. Ewald, *Ann. Phys.*, 1921, 369, 253-287. b) M. P. Tosi, *Solid State Phys.*, 1964, 16, 1-120.

[8] T. Kongkhlang, K. Tashiro, M. Kotaki, S. Chirachanchai, *J. Am. Chem. Soc.* 2008, 130, 15460.

[9] G. Zerbi, P. Hendra, *J. Mol. Spectrosc.* 1968, 27, 17.

[10] H. Dua, Y. X. Yin, X. X. Zeng, J. Y. Li, J. L. Shi, Y. Shi, R. Wen, Y. G. Guo, L. J. Wan, *Energy Storage Mater.* 2017, S2405829717303525.

[11] N. Voigt, L. van Wüllen, *Solid State Ionics* 2014, 260, 65.

[12] J. Chai, Z. Liu, J. Ma, J. Wang, X. Liu, H. Liu, J. Zhang, G. Cui, L. Chen, *Adv. Sci.* 2017, 4, 1600377.

[13] Q. Zhao, X. Liu, S. Stalin, K. Khan, L. A. Archer, *Nat. Energy* 2019, 4, 365.

[14] J. Zhou, T. Qian, J. Liu, M. Wang, L. Zhang, C. Yan, *Nano Lett.* 2019, 19, 3066.

[15] S. Huang, Z. Cui, L. Qiao, G. Xu, J. Zhang, K. Tang, X. Liu, Q. Wang, X. Zhou, B. Zhang, *Electrochim. Acta* 2019, 299, 820.
